# Later menopause confers no additional protection against osteoporosis in older women

**DOI:** 10.1530/EC-26-0078

**Published:** 2026-07-07

**Authors:** Zhiyi Zhou, Hao Li, Zhaojun Lu, Jiarui Chen, Jiang Xue, Siqing Song, Dequan Liu, Yufan Xu, Xinli Zhan, Chong Liu

**Affiliations:** The First Affiliated Hospital of Guangxi Medical University, Nanning, Guangxi, China

**Keywords:** osteoporosis, postmenopause, health surveys, estrogen, hormone replacement therapy

## Abstract

**Graphical Abstract:**

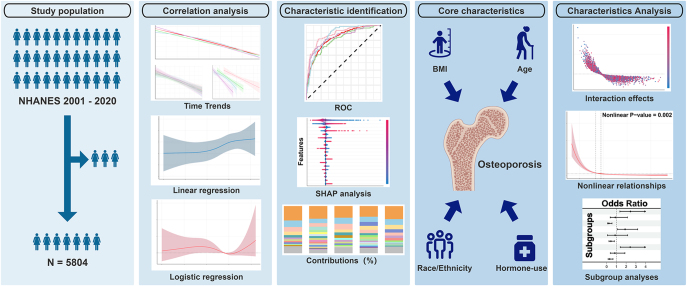

**Abstract:**

**Background**

Previous studies may have overestimated the effect of age at menopause when assessing osteoporosis risk and may have overlooked women with late menopause as a high-risk group. Therefore, we aim to clarify the association between age at menopause and osteoporosis risk, as well as to identify key characteristics of individuals with osteoporosis.

**Methods**

This study utilized a nationally representative cross-sectional sample, including 5,804 postmenopausal women aged 55–79 years. Weighted multivariate linear regression and logistic regression models were used to assess the association between age at menopause and femoral neck BMD and osteoporosis. Core characteristics of the osteoporosis population were identified by calculating standardized coefficients and applying SHapley Additive exPlanations (SHAP) analysis. Restricted cubic spline (RCS) analysis and subgroup analysis were conducted on these core characteristics.

**Results**

The age at menopause showed no significant association with osteoporosis risk in any model (*P* > 0.05). The two primary modifiable factors associated with reduced osteoporosis risk were BMI (OR = 0.84, 95% CI: 0.80–0.88) and hormone-use history (OR = 0.50, 95% CI: 0.33–0.74). Furthermore, RCS analyses revealed significant nonlinear relationships between age at menopause and both BMI (*P* < 0.001) and hormone-use history (*P* < 0.001). Subgroup analyses indicated that the compensatory effects of these two factors varied across different specific subgroups.

**Conclusion**

This study reveals that the initial protective advantage in reducing osteoporosis risk conferred by a later age at menopause diminishes in later life. Maintaining a higher BMI and appropriate hormone therapy can effectively compensate for the initial risk disadvantage.

## Introduction

Osteoporosis is a systemic skeletal disorder characterized by reduced bone mass and impaired bone microarchitecture, leading to increased bone fragility and elevated fracture risk (1). With the intensifying global aging population, this disease has become a major health threat and will continue to intensify (2). A study indicates that the global prevalence of osteoporosis is estimated to be 18.3%, with a prevalence rate of 23.1% among women (3). Bone loss in women accelerates dramatically following menopause, primarily driven by the sharp decline in estrogen levels, which triggers systemic abnormalities in bone metabolism (4, 5). This gender disparity places a disproportionate disease burden on women, encompassing substantial economic costs and profound psychological impacts, and directly leads to the definition of ‘postmenopausal osteoporosis’ (6, 7).

For many years, age at menopause has been regarded as a key indicator for assessing women’s long-term bone health (1, 7). Numerous prospective cohort studies have confirmed its association with osteoporosis: earlier age at menopause is associated with a higher long-term risk of osteoporosis (8, 9, 10). However, because the most rapid phase of postmenopausal bone loss occurs within 5–10 years after menopause (11), the design of these studies may have introduced selection bias or confounding. First, the age window was selected too early, resulting in study populations that included both premenopausal and postmenopausal women, or women at different stages of rapid bone loss. This effectively meant that these studies compared differences in menopausal status or duration of postmenopausal exposure, rather than the effect of age at menopause itself. Second, using chronological age as the time scale confounded the effect of age at menopause with the effect of time since menopause. Although women with earlier menopause may reach osteoporosis sooner due to longer cumulative exposure, those with later menopause may experience a steeper rate of bone loss. Therefore, when the analytical focus shifts from the early postmenopausal period to late postmenopause – a stage in which all individuals have passed the rapid phase of postmenopausal bone loss and osteoporosis becomes highly prevalent (12) – it warrants reexamination of whether age at menopause itself remains a hallmark risk characteristic.

To address this knowledge gap, this study, based on a nationwide cross-sectional survey, aims to i) elucidate the association between age at menopause and the risk of osteoporosis among postmenopausal older women and ii) identify key characteristics associated with osteoporosis in this population, particularly those that are modifiable. The findings will help determine whether the protective effect of later menopause persists into late life and, more importantly, will identify modifiable factors that could serve as actionable targets for personalized prevention in older postmenopausal women.

## Methods

### Data source and study population

All data utilized in this study were obtained from the National Health and Nutrition Examination Survey (NHANES). The NHANES is a series of cross-sectional national surveys administered by the National Center for Health Statistics (NCHS), a division of the Centers for Disease Control and Prevention (CDC), using a stratified multistage probability sampling design to assess the health and nutritional status of the civilian population in the United States (13). Data from the NHANES are collected through face-to-face interviews, standardized physical examinations, and laboratory testing of blood and urine samples, with survey results released every two years. All research protocols were approved by the National Center for Health Statistics Research Ethics Review Board (14). For more project details, visit the NHANES official website (https://www.cdc.gov/nchs/nhanes/index.htm).

The data for this study were derived from nine cycles of the NHANES from 2001 to 2020. We selected women aged 55–79 years with complete bone mineral density (BMD) data. The lower age limit of 55 years was set to encompass a sufficiently large sample of the elderly population and align with clinical guidelines recommending screening for women aged ≥50 years (1, 15). The upper limit of 79 years was chosen to avoid potential truncation bias arising from NHANES’s standardized recording of age ≥80 years. We determined each participant’s menopausal age through the questionnaire item: ‘How old were you when you had your last menstrual period?’. We established the inclusion criterion as ‘age at interview minus age at menopause >1 year’. Exclusion criteria included the following: missing data on age at menarche or menopause, postmenopausal status <1 year, pregnancy or lactation, and abnormal age at menarche (<8 years) or menopause (>65 years). Ultimately, 5,804 participants were included in the analysis, evenly distributed across survey cycles (Fig. S1 (see section on Supplementary materials given at the end of the article)).

### Outcome

This study designated femoral neck BMD as the primary outcome measure. This selection was based on two considerations. First, the femoral neck is one of the most common sites for osteoporotic fractures and carries the poorest prognosis (1, 16). Second, BMD at femoral neck is more sensitive to factors such as hormonal changes, medications, metabolic status, and mechanical loading, and it exhibits a relatively faster rate of loss (17, 18). All BMD measurements were obtained using dual-energy X-ray absorptiometry (DXA), which has become the gold standard for clinical BMD assessment due to its operational convenience, rapid scanning, and low radiation exposure.

The diagnosis of osteoporosis is based on the criteria recommended by the World Health Organization (WHO) in 1994 (19), using the T-score of femoral neck BMD. The T-score is calculated as the difference between an individual’s measured BMD value and the mean BMD of a reference population of healthy non-Hispanic white women aged 20–29 years, divided by the standard deviation (SD) of that reference population (T-score = (measured value – reference mean)/reference SD) (20). The reference data originate from the third National Health and Nutrition Examination Survey (NHANES III) in the United States (21). According to the WHO definition, a T-score ≤ −2.5 SD indicates osteoporosis, while a T-score between −2.5 and −1.0 indicates osteopenia. This study grouped subjects based on these criteria.

### Study variables

Drawing on previous research (4, 10, 22, 23), clinical guidelines (1, 15, 24), and data available from NHANES, the study variables include demographic factors (age, race/ethnicity, and marital status), socioeconomic factors (education level and poverty income ratio (PIR)), lifestyle factors (smoking, drinking, physical activity, and BMI), laboratory test data (level of 25-hydroxyvitamin D3 (VD3) and alkaline phosphatase (ALP)), obstetric and gynecological history factors (age at menarche, counts of pregnancy and delivery, and history of hysterectomy and ovariectomy), disease-related factors (diabetes, osteoarthritis, rheumatoid arthritis, and chronic kidney disease (CKD)), and medication-related factors (history of using hormones, glucocorticoids, and dietary supplements). Data for these variables were obtained through examinations or questionnaires, as detailed in Table S1.

### Statistical analysis

In the baseline data, continuous variables are represented by weighted means (weighted SD), while categorical variables are described by unweighted case counts (weighted percentages). For variables with a missing rate below 20%, we employed multiple imputation by chained equations for data imputation (25). Group comparisons were conducted using weighted analysis of variance (for continuous variables) or weighted chi-square tests (for categorical variables), with corresponding *P*-values calculated.

To visualize the age-BMD relationship across menopausal age subgroups in this cross-sectional sample, we fitted a generalized additive model (GAM) with sampling weights. The 95% confidence intervals (CIs) were derived from 1,000 nonparametric bootstrap iterations to quantify estimation uncertainty. It is critical to note that these curves depict the average BMD differences across ages among the surviving participants at the time of the survey. Consequently, the curves should not be interpreted as representing the true longitudinal rate of individual bone loss. Rather than focusing on mean group differences, our analysis compares the estimated prevalence of high or low BMD at each age across subgroups. This visualization serves to describe and compare age-BMD distribution patterns within the extant population, thereby generating hypotheses for future longitudinal research.

We employed weighted linear regression and weighted logistic regression to assess the associations of age at menopause with BMD and osteoporosis prevalence, respectively. These associations were evaluated across five sequentially adjusted models: Model 1 was unadjusted; Model 2 was adjusted for demographic, socioeconomic, and lifestyle factors; Model 3 further incorporated obstetric and gynecological history; Model 4 added disease- and medication-related factors; and Model 5 included all aforementioned covariates plus laboratory test results. Regression coefficients (β) and odds ratios (ORs) with their 95% confidence intervals (CIs) were calculated for each model.

Model discrimination was assessed using the receiver operating characteristic (ROC) curve. Subsequently, the SHapley Additive exPlanations (SHAP) method was employed to interpret the logistic regression model and rank variable importance. SHAP, rooted in cooperative game theory, robustly quantifies a feature’s contribution by calculating the average marginal effect of including that variable across all possible subsets of other features (26). We chose logistic regression for its inherent clinical interpretability and to ensure consistency with our planned advanced analyses. For comparative purposes, standardized coefficients were also calculated as a traditional, though less rigorous, supplementary measure of variable importance.

In addition, potential nonlinear relationships were examined using a restricted cubic spline (RCS) regression model with four knots. Subgroup analyses were performed based on key demographic and clinical characteristics to assess potential interactions. Given that weighted data may result in overly wide confidence intervals, we conducted our analyses using unweighted data in the variable importance analysis, RCS analysis, and subgroup analysis. To evaluate the robustness of the primary findings, sensitivity analyses were conducted by varying the inclusion criteria, utilizing unweighted data, and altering the outcome definitions. All statistical analyses and visualizations were performed using R (version 4.5.2). A two-sided *P*-value <0.05 was considered statistically significant.

## Results

### Baseline characteristics

Table S2 presents the baseline characteristics of the study population stratified by femoral neck T-score categories (normal, osteopenia, and osteoporosis). As shown in Table S2, this study included 5,804 participants, representing 22,423,228 elderly postmenopausal women after weighting calculations. The osteoporosis and osteopenia groups were generally associated with older age, specific ethnicities (non-Hispanic white and others), being unmarried, lower education and income, lower BMI, smoking, higher alcohol intake, reduced physical activity, older age at menarche, longer postmenopausal years, a history of gynecological surgery, diabetes, chronic kidney disease (CKD), non-use of hormone, glucocorticoid use, and lower vitamin D3 (VD3) levels, aligning with known risk profiles (1, 7). A notable exception was the lack of a significant difference in age at menopause across groups.

Table 1 presents the baseline characteristics of participants stratified by quartiles of age at menopause. Notably, the groups were well balanced in age (*P* = 0.453), effectively mitigating age as a major confounder in subsequent analyses of BMD. Significant differences were observed across quartiles for race/ethnicity, marital status, education, income, BMI, smoking, and annual drinking frequency (all *P* < 0.05), indicating a clear association between menopausal age and sociodemographic factors. Furthermore, the prevalence of hysterectomy and ovariectomy differed significantly (all *P* < 0.001), suggesting iatrogenic factors may be an important driver of premature menopause. Significant variations were also noted in serum VD3 levels, prevalence of diabetes, rheumatoid arthritis, CKD, and the history of hormone use (all *P* < 0.05), pointing to distinct comorbidity profiles and medication histories. Most importantly, and consistent with our prior findings, neither femoral neck T-scores nor the prevalence of osteoporosis differed significantly across menopausal age groups (all *P* > 0.05), reinforcing that menopausal age itself may not be a key determinant of osteoporosis in these elderly postmenopausal women.

**Table 1:** Baseline characteristics of participants from NHANES (2001–2020).

Characteristics	Grouped by quartiles of age at menopause					*P*-value
	Overall	Q1	Q2	Q3	Q4	
Sample size	5,804	1,490	1,653	1,509	1,152	
Weighted population	22,423,228	5,462,343	6,168,330	5,966,345	4,826,210	
Age at menopause (year)	46.10 (± 8.03)	34.43 (± 5.03)	45.14 (± 2.11)	50.53 (± 1.06)	55.06 (± 1.95)	**<0.001**
Age (year)	64.26 (± 6.79)	64.67 (± 6.77)	64.42 (± 6.97)	63.67 (± 6.85)	64.33 (± 6.43)	0.453
Race/ethnicity						**<0.001**
Mexican American	885 (4.2%)	225 (4.2%)	280 (5.0%)	244 (4.5%)	136 (2.9%)	
Non-Hispanic black	1,269 (9.7%)	392 (12.2%)	351 (10.4%)	294 (8.5%)	232 (7.6%)	
Non-Hispanic white	2,735 (76.9%)	696 (75.4%)	757 (75.3%)	703 (76.7%)	579 (80.8%)	
Others	915 (9.2%)	177 (8.2%)	265 (9.3%)	268 (10.3%)	205 (8.7%)	
Marital status						**0.021**
Married	3,069 (60.0%)	736 (57.3%)	866 (57.9%)	809 (61.3%)	658 (63.9%)	
Unmarried	2,735 (40.0%)	754 (42.7%)	787 (42.1%)	700 (38.7%)	494 (36.1%)	
Education						**<0.001**
Below high school	1,585 (16.8%)	460 (22.4%)	466 (17.4%)	383 (14.2%)	276 (13.0%)	
High school or above	4,219 (83.2%)	1,030 (77.6%)	1,187 (82.6%)	1,126 (85.8%)	876 (87.0%)	
PIR						**<0.001**
Poor	1,494 (16.2%)	454 (20.6%)	431 (17.3%)	364 (14.2%)	245 (12.5%)	
Not poor	4,310 (83.8%)	1,036 (79.4%)	1,222 (82.7%)	1,145 (85.8%)	907 (87.5%)	
BMI (kg/m^2^)	29.58 (± 6.86)	30.51 (± 7.01)	29.31 (± 6.88)	29.16 (± 6.69)	29.40 (± 6.80)	**<0.001**
Smoking						**<0.001**
Smoking	810 (14.0%)	284 (18.5%)	233 (15.2%)	187 (12.8%)	106 (9.1%)	
Not smoking	4,994 (86.0%)	1,206 (81.5%)	1,420 (84.8%)	1,322 (87.2%)	1,046 (90.9%)	
Drinking counts	47.92 (± 95.04)	34.77 (± 82.04)	42.98 (± 88.24)	57.40 (± 105.12)	57.42 (± 101.62)	**0.043**
Physical activity						0.319
High	1,101 (22.3%)	265 (21.9%)	308 (21.7%)	294 (23.0%)	234 (22.7%)	
Medium	2,155 (38.3%)	527 (36.4%)	615 (39.9%)	557 (36.1%)	456 (36.4%)	
Low	2,548 (39.4%)	698 (41.7%)	730 (38.4%)	658 (40.9%)	462 (40.9%)	
Obstetric and gynecological history						
Age at menarche (year)	12.79 (± 1.67)	12.70 (± 1.71)	12.71 (± 1.66)	12.89 (± 1.57)	12.85 (± 1.75)	0.982
Postmenopausal years	18.16 (± 10.56)	30.24 (± 8.17)	19.28 (± 7.33)	13.14 (± 6.94)	9.27 (± 6.16)	**<0.001**
Pregnancy counts	3.15 (± 2.15)	3.19 (± 2.12)	3.15 (± 2.16)	3.21 (± 2.28)	3.01 (± 2.01)	0.684
Delivery counts	2.50 (± 1.79)	2.61 (± 1.79)	2.50 (± 1.85)	2.47 (± 1.77)	2.41 (± 1.71)	0.186
Hysterectomy	2,460 (41.7%)	1,200 (83.4%)	773 (47.2%)	302 (19.1%)	185 (15.4%)	**<0.001**
Ovariectomy	1,520 (26.5%)	625 (44.2%)	535 (34.5%)	224 (13.9%)	136 (11.9%)	**<0.001**
Comorbidities						
Diabetes	1,178 (15.5%)	351 (19.2%)	345 (16.1%)	279 (12.7%)	203 (14.0%)	**<0.001**
Osteoarthritis	1,328 (27.3%)	371 (28.7%)	358 (27.7%)	347 (27.6%)	252 (25.1%)	0.498
Rheumatoid arthritis	567 (7.6%)	186 (10.2%)	143 (7.0%)	130 (6.2%)	108 (7.1%)	**0.004**
CKD	217 (3.1%)	85 (4.4%)	58 (3.4%)	45 (2.5%)	29 (1.9%)	**0.018**
History of medications/dietary supplements						
Hormones	2,374 (47.9%)	767 (59.5%)	713 (51.1%)	486 (38.3%)	408 (42.4%)	**<0.001**
Glucocorticosteroids	357 (6.1%)	108 (6.5%)	97 (6.0%)	87 (6.2%)	65 (5.6%)	0.868
Dietary supplements	2,426 (46.9%)	606 (46.0%)	670 (45.9%)	621 (47.3%)	529 (48.7%)	0.679
VD3 (nmol/L)	65.71 (± 26.36)	62.78 (± 27.05)	65.42 (± 26.29)	66.88 (± 27.46)	67.94 (± 23.84)	**0.035**
ALP (IU/L)	74.83 (± 24.45)	76.38 (± 25.21)	74.70 (± 26.04)	74.48 (± 21.70)	73.69 (± 24.64)	0.105
Femoral neck T-score	−0.87 (± 0.97)	−0.86 (± 0.98)	−0.89 (± 0.97)	−0.86 (± 0.97)	−0.87 (± 0.99)	0.892
Categorized by T-score	0.284					
Normal	3,475 (59.2%)	916 (61.2%)	974 (58.9%)	910 (58.7%)	675 (57.9%)	
Osteopenia	2,035 (35.9%)	486 (32.8%)	598 (36.3%)	537 (37.3%)	413 (37.1%)	
Osteoporosis	295 (4.9%)	88(6.0%)	81 (4.8%)	62 (4.0%)	64 (5.0%)	

PIR, poverty income ratio; BMI, body mass index; CKD, chronic kidney disease; VD3, 25-hydroxyvitamin D3; ALP, alkaline phosphatase; BMD, bone mineral density; NHANES, National Health and Nutrition Examination Survey; SD, standard deviation.

Characterization statistics are presented as weighted mean (± weighted SD) or unweighted sample size (*n*) (weighted %).

*P*-values for continuous variables were calculated using weighted analysis of variance (ANOVA), and *P*-values for categorical variables were calculated using weighted chi-square tests. Bold values indicate *P* < 0.05.

### Visualization of age-specific BMD distributions by groups

**Figure 1:**
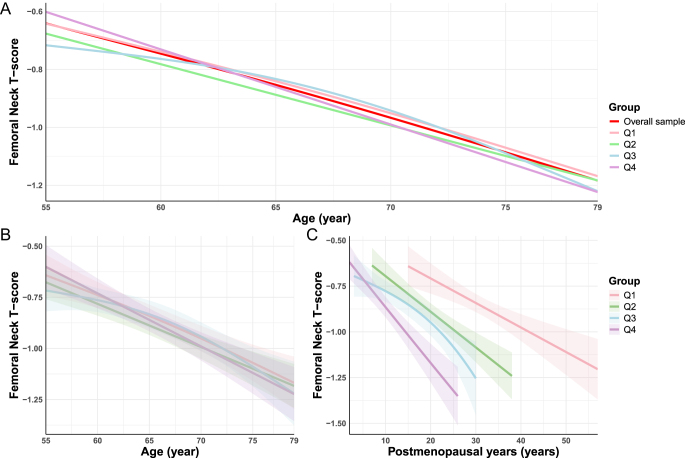
Trend of femoral neck T-scores over time. Curves were estimated using weighted generalized additive models (weighted GAM) fitted to scatter plots from each group, with shaded areas indicating 95% CI. (A) Overall and group trends in femoral neck T-scores by age. (B) Trends in femoral neck T-scores by age with 95% CI for each subgroup. (C) Trends in femoral neck T-scores by postmenopausal years with 95% CI for each group.

Figure S2 depicts the unadjusted relationship between age and femoral neck BMD in the overall population. The trend curve, derived from a weighted generalized additive model (GAM) without adjustment for confounders, reflects the real-world clinical trajectory of femoral neck T-scores, showing a continuous decline with age that aligns with established physiological patterns and previous epidemiological observations (27). Using the same method, we generated age–BMD curves for the population stratified by menopausal age quartiles, as summarized in Fig. 1.

At age 55, the Q4 group (latest menopause) exhibited the highest BMD (Fig. 1A), consistent with the estrogen-protection hypothesis. However, the Q1 group (earliest menopause) did not show the lowest BMD, suggesting that early menopausal women may not have the frailest bones as conventionally expected. With advancing age, the BMD of the Q4 group declined to the lowest level among all groups by age 79, whereas the Q1 group maintained a relatively higher BMD. The substantial overlap in the 95% CI in Fig. 1B quantitatively confirms the reason for the limited differences between groups: excessive variation in individual BMD. This implies that age at menopause may not be the variable very strongly associated with BMD. Furthermore, the trajectories of BMD decline differed across groups (Fig. 1C), with Group Q4 exhibiting the steepest decline curve due to being in the postmenopausal phase of rapid bone loss, while other groups showed a more gradual decline as they had passed this phase. This suggests a potential compensatory mechanism, whereby late menopause may not confer a long-term protective advantage but could instead be linked to an accelerated rate of bone loss in later life, while early menopause may initiate adaptive processes that lead to a more gradual net bone loss over time.

### Association between age at menopause and BMD as well as prevalent osteoporosis

Using weighted linear and logistic regression models adjusted for potential confounders, we analyzed the associations of age at menopause with femoral neck T-scores (Table S3) and osteoporosis probability (Table 2), respectively. Age at menopause was not a significant predictor, whether modeled as a continuous variable or in quartiles, with all regression coefficients (β) and odds ratios (ORs) being non-significant (all *P* > 0.05). Analysis of the fully adjusted model (Model 5) identified several significant predictors of osteoporosis risk (Fig. S3): age (OR = 1.09, 95% CI: 1.06–1.12); BMI (OR = 0.84, 95% CI: 0.80–0.88); race/ethnicity (ref: non-Hispanic black), non-Hispanic white (OR = 4.32, 95% CI: 2.50–7.46), Mexican American (OR = 2.57, 95% CI: 1.34–4.94), and other races/ethnicities (OR = 5.76, 95% CI: 3.23–10.27); history of hormone use (OR = 0.50, 95% CI: 0.33–0.74); ALP levels (OR = 1.01, 95% CI: 1.00–1.01); VD3 levels (OR = 0.99, 95% CI: 0.98–1.00); and smoker (OR = 1.59, 95% CI: 1.04–2.41).

**Table 2:** Association between age at menopause and osteoporosis risk.

Age at menopause (years)	Model 1		Model 2		Model 3		Model 4		Model 5	
	OR (95%CI)	*P*-value	OR (95%CI)	*P*-value	OR (95%CI)	*P*-value	OR (95%CI)	*P*-value	OR (95%CI)	*P*-value
Continuous variable	0.992 (0.975, 1.008)	0.312	0.998 (0.982, 1.015)	0.840	0.995 (0.977, 1.014)	0.625	0.995 (0.977, 1.014)	0.628	0.995 (0.973, 1.017)	0.655
Grouped variable (median (Q1, Q3))										
Q1 (35 (32, 39))	Reference		Reference		Reference		Reference		Reference	
Q2 (45 (44, 47))	0.778 (0.532, 1.137)	0.196	0.818 (0.553, 1.211)	0.317	0.762 (0.509, 1.142)	0.190	0.749 (0.498, 1.128)	0.169	0.665 (0.421, 1.052)	0.084
Q3 (50 (50, 52))	0.650 (0.427, 0.989)	0.046	0.733 (0.471, 1.140)	0.170	0.655 (0.401, 1.070)	0.094	0.628 (0.381, 1.035)	0.070	0.590 (0.336, 1.037)	0.069
Q4 (55 (54, 56))	0.809 (0.529, 1.238)	0.331	0.948 (0.606, 1.485)	0.817	0.833 (0.497, 1.395)	0.489	0.835 (0.502, 1.389)	0.489	0.820 (0.472, 1.426)	0.484
*P* for trend	0.126		0.477		0.298		0.274		0.310	

Model 1: did not adjusted for relevant covariates. Model 2: adjusted for age, race/ethnicity, marital status, education, PIR, smoking, drinking, and physical activity. Model 3: further adjusted for age at menarche, pregnancy, delivery, hysterectomy, and ovariectomy. Model 4: further adjusted for diabetes, osteoarthritis, rheumatoid arthritis, CKD, hormones, glucocorticoids, and dietary supplements. Model 5: further adjusted for BMI, VD3, and ALP.

*P*-values for trend were calculated by modeling the median values of each quantile.

### Variable importance analysis

**Figure 2:**
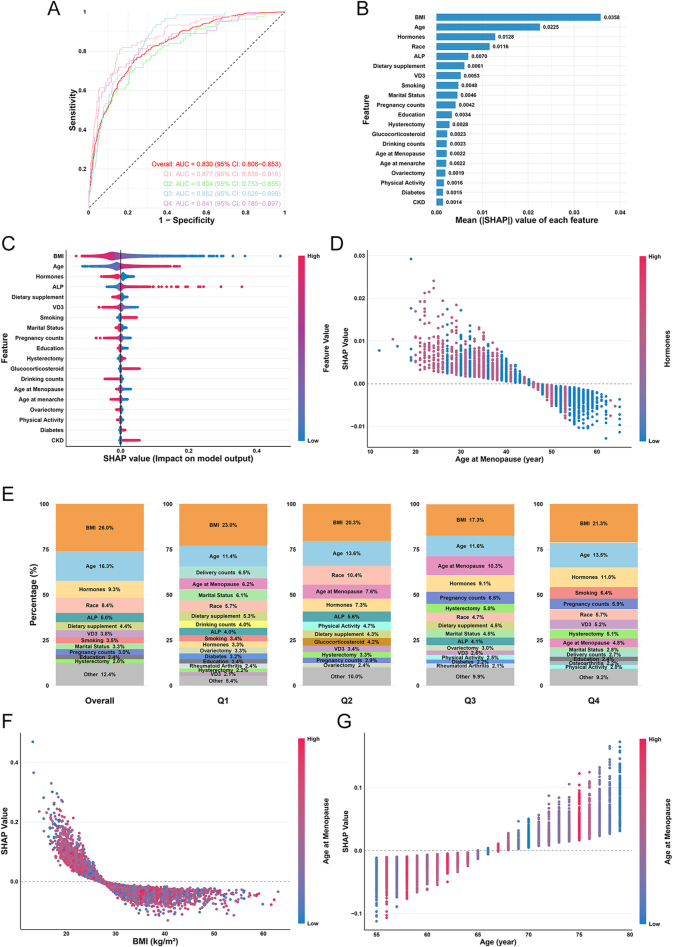
Visualization of SHAP analysis. (A) Receiver operating characteristic (ROC) curves for the model across all groups. (B) Top 20 variables ranked by SHAP values. (C) SHAP summary plot. (D) SHAP scatter plot of age at menopause. (E) Relative SHAP importance of each variable. (F) SHAP scatter plot of the interaction effect between BMI and age at menopause. (G) SHAP scatter plot of the interaction effect between hormone-use status and age at menopause. Analysis based on the fully adjusted logistic regression model (Model 5).

To assess variable importance, we first evaluated the model’s robustness across different menopausal age subgroups using the area under the receiver operating characteristic curve (AUC). The model demonstrated excellent discriminatory ability, with an overall AUC of 0.830 (95% CI: 0.806–0.853), and all subgroups achieved AUC values exceeding 0.8 (Fig. 2A).

Variable importance was subsequently ranked using two methods: standardized coefficients (Fig. S4A) and SHapley Additive exPlanations (SHAP) values (Fig. 2B). Both methods consistently identified BMI, age, hormone-use history, and race/ethnicity as the top four most influential characteristics, collectively accounting for over 60% of the total contribution (Fig. 2E). Although ALP and VD3 levels were also statistically significant, their relatively lower effect sizes (Fig. S3) led to their exclusion from the core set of top predictors. Among the core characteristics, age emerged as a positive risk factor, whereas BMI and hormone-use history acted as protective factors (Fig. 2C) and were modifiable factors.

Further quantification of relative contributions (Figs 2E and S4B) revealed BMI and age as the most stable and dominant characteristics. Racial influence showed minor fluctuations but remained generally stable. Notably, the impact of hormone-use history gradually increased with delayed menopause age, suggesting that its protective effect may be more pronounced in women with later menopause. Age at menopause itself showed lower significance in standardized coefficient analysis but ranked higher in SHAP analysis, which accounts for marginal and interaction effects. However, its absolute contribution still decreased with increasing menopause age, suggesting that its influence may manifest indirectly through other variables and gradually diminish with advancing menopause age.

Finally, SHAP dependency plots revealed specific interactions: a significant association between age at menopause and hormone-use history (Fig. 2D), manifesting as lower hormone-use rates among women with older age at menopause. Conversely, no such interaction was detected for BMI or age (Fig. 2F and G). This finding was validated through formal interaction analysis (Fig. S5).

### Restricted cubic spline (RCS) analysis

**Figure 3:**
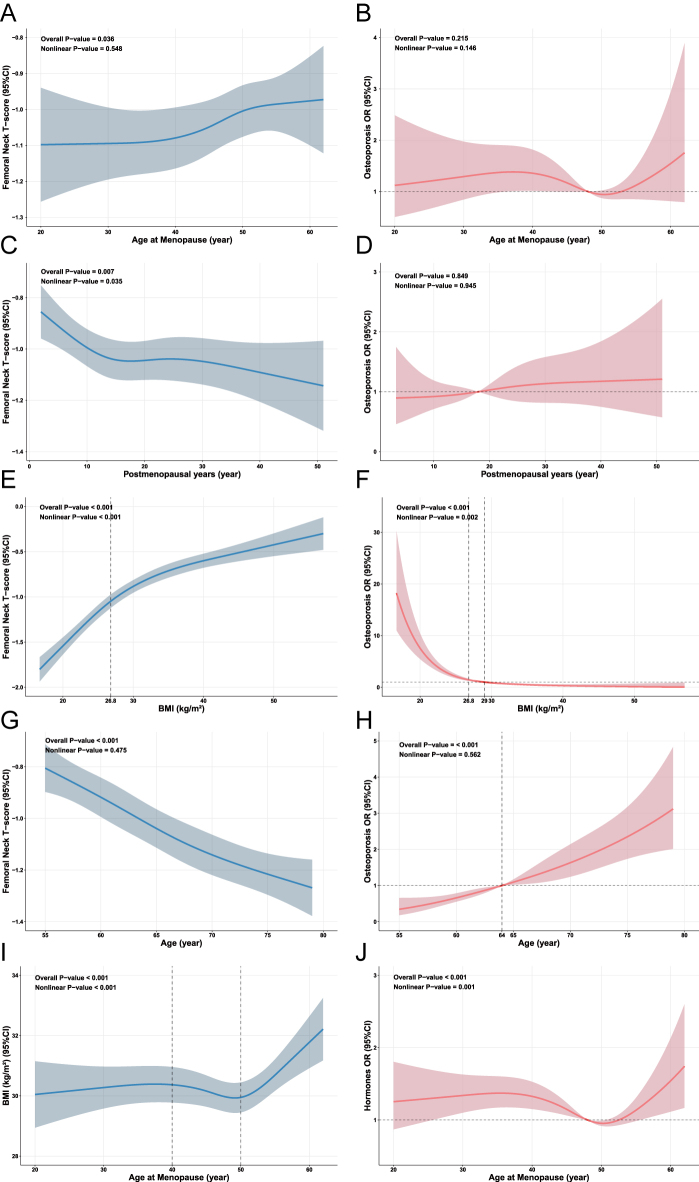
Restricted cubic spline (RCS) plot matrix. (A, B, C, D, E, F, G, H, I, J) Dose–response relationships between different exposures and outcomes: (A) Age at menopause and femoral neck T-score; (B) Age at menopause and osteoporosis risk; (C) Postmenopausal years and femoral neck T-score; (D) Postmenopausal years and osteoporosis risk; (E) BMI and femoral neck T-score; (F) BMI and osteoporosis risk; (G) Age and femoral neck T-score; (H) Age and osteoporosis risk; (I) Age at menopause and BMI; (J) Age at menopause and hormone-use status. All RCS curves are estimated based on multivariate models and uniformly adjusted for a preset set of covariates. In the figures, curves corresponding to continuous outcome variables are shown in blue, while those for categorical outcome variables are shown in red.

We employed an RCS model to explore potential nonlinear relationships between independent variables and outcome measures (femoral neck T-score or osteoporosis risk). The results indicate that although both age at menopause and postmenopausal years are associated with femoral neck T-scores (Fig. 3A and C), neither is significantly associated with the risk of osteoporosis (Fig. 3B and D). This suggests that the protective effects of late menopause or a short postmenopausal period may not be evident in older age. BMI exhibited significant nonlinear associations with both femoral neck T-score and osteoporosis risk (Fig. 3E and F). Concurrently, a cutoff point for osteoporosis risk was found to be at a BMI ≈29 kg/m^2^, suggesting that individuals may need to reach obesity levels to reduce their risk of osteoporosis. Furthermore, maximum curvature point analysis identified an inflection point at BMI = 26.8 kg/m^2^, indicating an accelerated rise in osteoporosis risk below this value, which warrants attention. Age showed a linear association with both femoral neck T-score and osteoporosis risk (Fig. 3G and H). The age risk cutoff point was 64 years, beyond which osteoporosis risk significantly increased.

We also examined the association trends between BMI and hormone-use history – two important modifiable factors – and age at menopause. Both demonstrated nonlinear relationships (Fig. 3I and J). The slope of the BMI curve indicates that before the menopausal age of 50, the curve fluctuates slightly but remains generally stable, after which it rises sharply. Hormone-use rates were higher in both the youngest and oldest age at menopause groups, exhibiting a U-shaped pattern. These patterns suggest that, in real-world settings, the impact of age at menopause on osteoporosis risk may be mediated through its complex associations with BMI and hormone use.

### Subgroup analysis

**Figure 4:**
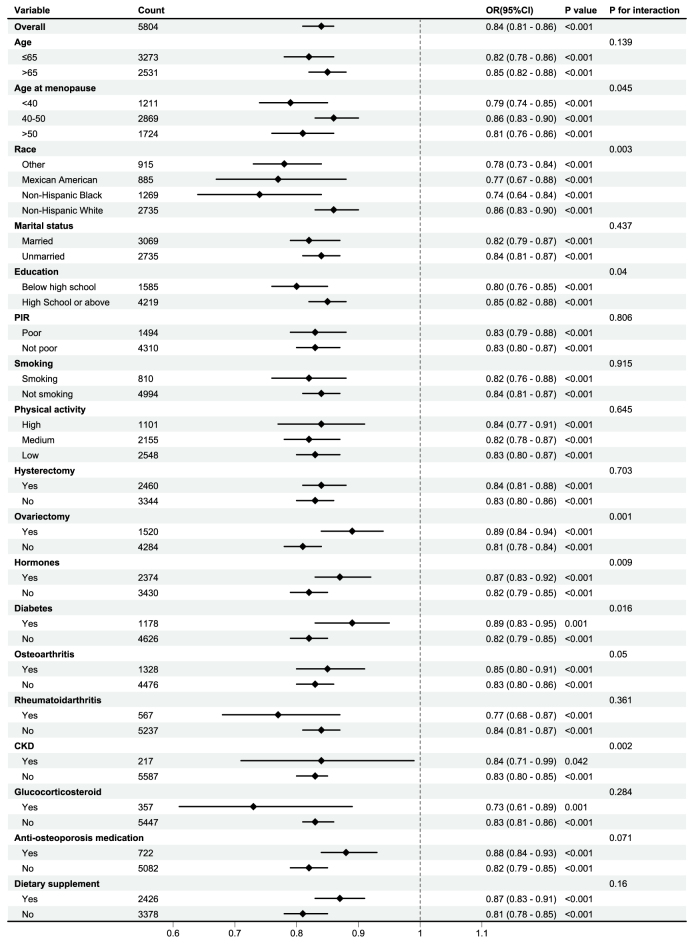
Subgroup analyses and forest plot of the association between BMI and osteoporosis risk. The estimates are based on a logistic regression model fully adjusted for all prespecified covariates. The centers of the diamonds in the figure correspond to the OR estimates for each subgroup. The horizontal lines extending from the diamond points indicate the 95% confidence intervals (CIs); the vertical dashed lines mark the null line OR = 1. The arrows indicate confidence intervals extending beyond the coordinate axes.

We conducted subgroup analyses based on BMI, age, hormone-use history, and age at menopause. Results showed that BMI and hormone-use history consistently demonstrated protective effects across all subgroups (Figs 4 and S6), whereas age consistently emerged as a risk factor in all subgroups (Fig. S7). Age at menopause showed no significant association with osteoporosis risk in any subgroup (Fig. S8). Notably, BMI exhibited significant interactions with multiple factors, indicating that its protective effect varies across different subgroups. Specifically, in certain subgroups (e.g. those with earlier age at menopause, lower education levels, or no history of hormone use), the protective effect of BMI was more pronounced among vulnerable individuals. Conversely, in other subgroups (e.g. specific ethnicities, those with a history of ovariectomy, or diabetes), higher BMI conferred stronger protective effects among advantaged individuals. Hormone-use history also exhibited similar, albeit weaker, effect-modifying roles. These findings suggest that for individuals at a disadvantage due to innate or early-life factors, postmenopausal management strategies – such as maintaining a healthy weight or hormone therapy – may help reduce osteoporosis risk.

### Sensitivity analyses

To validate the robustness of key findings, we conducted sensitivity analyses across three dimensions: statistical methods, study population, and outcome definitions. First, the association between age at menopause and femoral neck T-score, as well as osteoporosis risk, was re-evaluated in the unweighted sample. The results showed a positive correlation with femoral neck T-score (Table S4), but the association with osteoporosis risk remained statistically insignificant (Table S5). Second, after excluding participants with extreme menopausal ages (<30 years and >60 years), we reassessed the impact of each variable on osteoporosis risk. The effect sizes and directions of significance for each variable were consistent with the primary analysis results (Fig. S9). Finally, when the outcome was restricted to osteopenia (a precursor stage of osteoporosis), the patterns of association for BMI, age, and ethnicity remained substantially unchanged. Although the ranking of hormone-use history decreased slightly in importance, it remained a major influencing factor (Fig. S10). Collectively, these results indicate that the primary study conclusions are robust across different analytical strategies.

## Discussion

This study systematically evaluated the clinical characteristics and associated risk factors for osteoporosis in elderly postmenopausal women based on nationwide cross-sectional data. A key finding revealed no significant association between age at menopause and osteoporosis risk. Subsequent studies further confirmed that BMI, age, race/ethnicity, and history of hormone use are the most core characteristics associated with osteoporosis. In addition, as modifiable core factors, BMI, history of hormone use, and age at menopause exhibit complex nonlinear associations. Simultaneously, their protective effects demonstrate heterogeneity across different subgroups.

In our association analysis, we observed no statistically significant overall association between age at menopause and osteoporosis risk in elderly postmenopausal women. This indicates that after all study participants have undergone the period of rapid BMD decline caused by the postmenopausal drop in estrogen levels, the age at menopause has no significant impact on the risk of osteoporosis among individuals of the same age. Although it remains inconsistent with the majority of research conclusions (8, 9, 10) based on the ‘estrogen protection hypothesis’, which posits that delayed menopause maintains BMD by prolonging estrogen exposure (5). However, another study found no significant difference in BMD at old age between women who had early menopause and those who had late menopause (28). Our hypothesis stems from differences in the life stages of the study populations. The independent protective effect of menopausal age may cease during the perimenopausal period, as the most rapid bone loss typically occurs within 5–10 years postmenopause (11, 28, 29, 30). When study cohorts are confined to the 55–79 age range, the long-term effects of early menopause may be diluted by subsequent decades of age-related bone loss (31, 32).

Research has found that women who experience late menopause lose bone mass more rapidly after menopause than those who experience early menopause (33), which is consistent with our observations (Fig. 1C). Although late menopause prolongs the duration of endogenous estrogen exposure, it also means that the sharp decline in estrogen occurs at an older age, by which time the effects of aging have already begun to manifest. With advancing age, senescent cells accumulate in bone tissue and secrete pro-inflammatory factors (the senescence-associated secretory phenotype, SASP), creating a catabolic microenvironment (34). Importantly, while estrogen deficiency and cellular senescence drive bone loss through distinct mechanisms (35, 36), estrogen can prevent – but not reverse – osteocyte and osteoblast senescence via pathways such as Usp10-dependent p53 degradation (37, 38, 39). Therefore, when a woman experiences late menopause, she faces a dual challenge: the sudden withdrawal of estrogen not only eliminates its direct anti-osteoporotic effects but also removes its anti-senescence protection, while preexisting senescent cells continue to accelerate bone loss through SASP-mediated inflammation. Studies have found that aging-related physiological changes are more pronounced in women with late menopause (40, 41). This convergence of mechanisms explains why the late-menopause group exhibited the steepest BMD decline (Fig. 1C). Concurrently, women with late menopause are less likely to use hormone therapy (Table 1). Collectively, these findings underscore that, despite delayed menopause, women entering old age remain a high-risk group requiring close monitoring.

It is noteworthy that although no significant association was observed between age at menopause and osteoporosis risk, a statistically significant association with BMD T-scores was detected, albeit with a very small effect size (Table S4 and Fig. 3A). This seemingly discrepant finding underscores the important distinction between statistical significance and clinical relevance. Specifically, while later menopause may be associated with a modest increase in BMD, this effect may be too small to significantly reduce the prevalence of osteoporosis among older women. From a clinical perspective, other factors may deserve greater attention than age at menopause.

Previous studies have found that the age at menopause is closely associated with many factors (42, 43). Our study similarly observed a nonlinear relationship between the two key modifiable factors – BMI and history of hormone use – and the age at menopause (Fig. 3G and H). However, given that there was no significant association between age at menopause and osteoporosis risk in any of the models – from the unadjusted Model 1 to the fully adjusted Model 5 (Tables 2 and S5) – we conclude that the strong protective effects of higher BMI and hormone use (Figs 4 and S6) are independent of age at menopause. This further underscores the clinical utility of these two modifiable factors as targets for interventions to prevent osteoporosis in postmenopausal women.

Our findings confirm that higher BMI and a history of hormone therapy (HT) are core modifiable protective factors for BMD, aligning with established literature (8, 9, 44, 45, 46). Mechanistically, both can be viewed as compensatory pathways to address postmenopausal estrogen deficiency. BMI’s protection arises from weight-bearing mechanical stimulation (15) and, crucially, the additional estrogen produced by adipose tissue. The latter mechanism likely underlies the pronounced benefit observed in postmenopausal women (47) and is supported by our subgroup analysis, in which BMI’s effect was strongest among those with earlier age at menopause or with no history of HT (Fig. 4). However, the benefit plateaus at higher BMI levels (Fig. 3F), likely due to countervailing metabolic and pro-inflammatory effects of adiposity (22). In contrast, HT provides direct and potent estrogenic protection, which is especially crucial for women with iatrogenic premature menopause (Fig. S6) (39). However, the decision to initiate HT requires an individualized risk–benefit assessment (48). Subgroup analysis observation is that, despite lower utilization, HT remains highly effective in women with later menopause (Figs S5 and S6), highlighting a potential intervention gap in this subgroup.

In our study, age and race/ethnicity are two unmodifiable core characteristics associated with osteoporosis. Traditionally, age has been considered the primary driver of BMD decline (1, 15, 24). However, in women, menopause represents a critical physiological turning point for bone loss (28, 29, 30). Research has found that the postmenopausal years are a better indicator of BMD variations across different populations than chronological age (10). Once all individuals have passed through the phase of rapid postmenopausal BMD loss and entered a plateau of slow decline, the direct effect of age on BMD may be significantly diminished. Concurrently, racial/ethnic differences in BMD have been documented, with non-Hispanic black women typically exhibiting higher average BMD than other groups (21, 27, 49). However, non-Hispanic black women still face significant disparities in socioeconomic status, access to healthcare resources (50), and the distribution of age at menopause (Table 1). Notably, research indicates that BMD variability within any racial group is far greater than the average differences between groups, and fracture risk may be comparable across populations (51). This suggests that when considering racial/ethnic labels, greater attention should be paid to the combined effects of underlying social and structural factors.

The strengths of this study are primarily reflected in the following three aspects. First, the research focuses on the specific population of postmenopausal elderly women. It is one of the few studies revealing that the age at menopause lacks an independent association with osteoporosis risk in this group, suggesting that the protective effect of age at menopause may diminish with increasing age. This provides new epidemiological evidence for the underlying physiological mechanisms. Second, data originate from the nationally representative large-scale NHANES database, encompassing diverse racial and socioeconomic backgrounds, enhancing the generalizability of findings. Finally, methodologically, we integrated traditional logistic regression with machine learning interpretability techniques (SHAP). This approach ensures clinical interpretability of the model while objectively identifying key predictors, forming cross-validation to enhance the robustness of conclusions.

Nevertheless, this study has several limitations. First, the cross-sectional design cannot capture the BMD trend curves of individual participants, which significantly undermines the predictive or intervention value of this study. Second, key variables such as age at menopause and hormone-use history relied on retrospective self-reporting, potentially introducing recall bias. Finally, the absence of critical details regarding hormone use – including initiation age, duration of use, specific types, and dosages – limits our ability to conduct more in-depth heterogeneity analyses of treatment effects. Future studies should validate and deepen these findings through prospective cohort designs and the collection of more comprehensive treatment information.

## Conclusion

This study, based on nationally representative data, reveals that the initial protective advantage in BMD conferred by a later age at menopause diminishes in later life. Women who experience menopause later remain at a high risk of osteoporosis as they advance in age. More importantly, we found that maintaining a higher BMI and appropriate hormone therapy can effectively compensate for the initial risk disadvantage associated with earlier menopause. This provides crucial evidence for developing precise, stratified prevention strategies for postmenopausal women in the elderly stages.

## Supplementary materials



## Declaration of interest

The authors declare that there is no conflict of interest that could be perceived as prejudicing the impartiality of the work reported.

## Funding

The present research was supported by i) the National Natural Science Foundation of China (Grant number: NSFC82360422) and ii) the First-Class Discipline Innovation-Driven Talent Program of Guangxi Medical University.

## Author contribution statement

ZZ conceived the study; designed the methodology; performed formal analysis, investigation, and visualization; curated the data; and wrote the original draft of the manuscript. HL and ZL designed the methodology and performed validation. JC and JX performed visualization. SS, DL, and YX performed investigation and data curation. CL and XZ supervised the study, administered the project, acquired the funding, provided resources, and reviewed and edited the manuscript.

## Ethics approval and consent to participate

This study followed the ethical principles of the Declaration of Helsinki and the International Ethical Guidelines for Health-related Research Involving Humans (CIOMS, 2016) for retrospective analysis of anonymized data.

All NHANES cycle data were approved by the Ethics Review Board (ERB) of the National Center for Health Statistics (NCHS) under the U.S. Centers for Disease Control and Prevention (CDC) (Protocol number: #98-12, #2005-06, #2011-17, #2018-01. For details, see https://www.cdc.gov/nchs/nhanes/about/erb.html). All participants signed written informed consent forms prior to undergoing household interviews and physical examination procedures.

## Consent for publication

All listed authors have read the final version of the manuscript and agree to its publication if accepted.

## Availability of data and materials

The data underlying this study are freely accessible to all researchers through the National Health and Nutrition Examination Survey (NHANES) database. The data can be obtained by visiting the following website: https://www.cdc.gov/nchs/nhanes/index.htm.
