# Supplementary material for: Later menopause confers no additional protection against osteoporosis in older women
Source: Endocr Connect. 2026 Jul 7;15(7):e260078. doi: 10.1530/EC-26-0078 (PMC13385196; doi:10.1530/EC-26-0078)
Supplement: Supplementary file 1 [file EC-26-0078_supplementary_materials.pdf]

Supplementary files

Content

Table S1 Definition and Classification of Covariates..2
Table S2 Baseline characteristics of participants from NHANES (2001- 2020)..5
Table S3 Associations of age at menopause with Femoral Neck T-score..7
Table S4 Associations of age at menopause with Femoral Neck T-score (unweighted)..8
Table S5 Association between age at menopause and osteoporosis risk (unweighted)..9
Fig. S1 Study population selection char..10
Fig. S2 Association between Age and Femoral Neck T-Score..11
Fig. S3 Odds ratios (ORs) for osteoporosis risk factors..12
Fig. S4 Visualization of standardized coefficient calculation..13
Fig. S5 Interaction effect plot..14
Fig. S6. Subgroup analyses of the association between hormone-use history and osteoporosis risk..15
Fig. S7. Subgroup analyses of the association between age and osteoporosis risk..16
Fig. S8. Subgroup analyses of the association between hormone-use history and osteoporosis risk..17
Fig. S9 Sensitivity analyses (excluding participants with extreme age at menopause)..18
Fig. S10 Sensitivity analyses (outcome was restricted to osteopenia)..19

Table S1 Definition and Classification of Covariates.

Covariate	Section of the NHANES	Text	Definition
Age (year)	Demographics data	Age in years of the participant at the time of screening.	Continuous variable.<br>Categorical variable for subgroup analysis.
Race		Recode of reported race and Hispanic origin information	Categorical variable:<br>1 = Mexican American,<br>2 = Non-Hispanic White,<br>3 = Non-Hispanic Black,<br>4 = Other (including Other Hispanic and multi-racial).
Marital status		Marital status.	Categorical variable:<br>1 = Married (including married and living with partner),<br>2 = Unmarried (including widowed, Divorced, separated, never married).
Education		What is the highest grade or level of school completed or the highest degree you have received?	Categorical variable:<br>1 = Below high school,<br>2 = High school or above.
PIR		A ratio of family income to poverty guidelines.	Categorical variable:<br>1 = Poor (≤ 1.3),<br>2 = Not poor（> 1.3）.
BMI (kg/m²)	Examination data	Body mass index.	Continuous variable.<br>Categorical variable for subgroup analysis.
VD3 (nmol/L)	Laboratory data	25-hydroxyvitamin D3.	Continuous variable.
ALP (IU/L)		Alkaline Phosphatase.	Continuous variable.
Smoking	Questionnaire data	Do you now smoke cigarettes?	Categorical variable:<br>0 = No,<br>1 = Yes.
Drinking counts		How often drink alcohol over past 12 moth?	Continuous variable.

Physical activity		Physical activity covariates are derived from the Global Physical Activity Questionnaire (GPAQ) interview data and calculated using metabolic equivalent (MET) scores.	Categorical variable:<br>1 = Low,<br>2 = Medium,<br>3 = High.
Age at menarche (year)		Age when first menstrual period occurred.	Continuous variable.
Pregnancy counts		How many times have been pregnant?	Continuous variable.
Delivery counts		How many deliveries live birth result?	Continuous variable.
Hysterectomy		Had a hysterectomy?	Categorical variable:<br>0 = No,<br>1 = Yes.
Ovariectomy		Had at least one ovary removed?<br>Had both ovaries removed?	Categorical variable:<br>0 = No,<br>1 = Yes.
Diabetes		Doctor told you have diabetes.	Categorical variable:<br>0 = No,<br>1 = Yes.
Osteoarthritis		Which type of arthritis was it?	Categorical variable:<br>0 = No(Psoriatic arthritis，Other，Refused),<br>1 = Yes(Osteoarthritis).
Rheumatoid arthritis			Categorical variable:<br>0 = No(Psoriatic arthritis，Other，Refused),<br>1 = Yes(Rheumatoid arthritis).
CKD		Ever told you had weak/failing kidneys.	Categorical variable:<br>0 = No,<br>1 = Yes.
Hormones		Ever use female hormones?	Categorical variable:<br>0 = No,<br>1 = Yes.
Glucocorticosteroid		Ever taken prednisone or cortisone daily.	Categorical variable:<br>0 = No,

			1 = Yes.
Dietary supplement	Dietary data	Dietary supplement for bone health.	Categorical variable:<br>0 = No,<br>1 = Yes.

Abbreviations: PIR, poverty income ratio. BMI, body mass index. CKD, chronic kidney disease. VD3, 25-hydroxyvitamin D3. ALP, alkaline phosphatase. BMD, bone mineral density. NHANES, National Health and Nutrition Examination Survey.

Table S2 Baseline characteristics of participants from NHANES (2001- 2020).

Characteristics	Grouped by Femoral Neck T-score				P-value
	Overall	Normal	Osteopenia	Osteoporosis	
**Sample size**	5,804	3475	2,034	295	
**Weighted population**	22,423,228	13,277,329	8,045,025	1,100,874	
**Age at menopause (year)**	46.10 (± 8.03)	45.98 (± 8.08)	46.38 (± 7.94)	45.57 (± 7.96)	0.210
**Age (year)**	64.26 (± 6.79)	63.54 (± 6.68)	64.94 (± 6.70)	67.94 (± 7.01)	**<0.001**
**Race/Ethnicity**					**<0.001**
Mexican American	885 (4.2%)	554 (4.1%)	305 (4.7%)	26 (3.0%)	
Non-Hispanic Black	1,269 (9.7%)	956 (12.6%)	292 (5.9%)	21 (2.9%)	
Non-Hispanic White	2,735 (76.9%)	1,560 (75.6%)	1,005 (78.6%)	170 (79.4%)	
Other	915 (9.2%)	405 (7.7%)	432 (10.8%)	78 (14.8%)	
**Marital status**					**0.005**
Married	3,069 (60.0%)	1,821 (59.9%)	1,116 (61.7%)	132 (48.1%)	
Unmarried	2,735 (40.0%)	1,654 (40.1%)	918 (38.3%)	163 (51.9%)	
**Education**					**<0.001**
Below high school	1,585 (16.8%)	977 (17.8%)	512 (14.3%)	96 (23.6%)	
High school or above	4,219 (83.2%)	2,498 (82.2%)	1,522 (85.7%)	199 (76.4%)	
**PIR**					**<0.001**
Poor	1,494 (16.2%)	899 (16.4%)	506 (14.7%)	89 (25.3%)	
Not poor	4,310 (83.8%)	2,576 (83.6%)	1,528 (85.3%)	206 (74.7%)	
**BMI (kg/m²)**	29.58 (± 6.86)	30.94 (± 7.12)	27.98 (± 5.89)	24.84 (± 5.55)	**<0.001**
**Smoking**					**<0.001**
Smoking	810 (14.0%)	457 (13.2%)	284 (14.0%)	69 (23.4%)	
Not smoking	4,994 (86.0%)	3,018 (86.8%)	1,750 (86.0%)	226 (76.6%)	
**Drinking counts**	47.92 (± 95.04)	44.40 (± 91.49)	53.94 (± 99.52)	46.46 (± 101.43)	**0.024**
**Physical activity**					**0.014**
High	1,101 (22.3%)	649 (21.0%)	407 (24.7%)	45 (21.1%)	
Medium	2,155 (38.3%)	1,246 (37.3%)	795 (39.8%)	114 (39.0%)	
Low	2,548 (39.4%)	1,580 (41.8%)	832 (35.4%)	136 (39.9%)	

Table S2 Baseline characteristics of participants from NHANES (2001- 2020).

Characteristics	Grouped by Femoral Neck T-score				P-value
	Overall	Normal	Osteopenia	Osteoporosis	
**Obstetric and Gynecological History**					
Age at menarche (year)	12.79 (± 1.67)	12.71 (± 1.61)	12.91 (± 1.74)	12.77 (± 1.75)	**0.004**
Postmenopausal years	18.16 (± 10.56)	17.57 (± 10.55)	18.56 (± 10.42)	22.38 (± 10.74)	**0.018**
Pregnancy counts	3.15 (± 2.15)	3.24 (± 2.24)	2.99 (± 2.02)	3.16 (± 1.94)	**<0.001**
Delivery counts	2.50 (± 1.79)	2.58 (± 1.84)	2.37 (± 1.69)	2.51 (± 1.74)	**<0.001**
Hysterectomy	2,460 (41.7%)	1,548 (43.4%)	790 (38.7%)	122 (43.2%)	0.051
Ovariectomy	1,520 (26.5%)	981 (28.6%)	468 (22.9%)	71 (28.2%)	**<0.001**
**Comorbidities**					
Diabetes	1,178 (15.5%)	797 (18.4%)	334 (10.9%)	47 (13.7%)	**<0.001**
Osteoarthritis	1,328 (27.3%)	784 (27.1%)	484 (28.2%)	60 (24.4%)	0.564
Rheumatoid arthritis	567 (7.6%)	350 (8.1%)	190 (6.8%)	27 (7.3%)	0.364
CKD	217 (3.1%)	125 (2.7%)	75 (3.3%)	17 (6.0%)	**0.044**
**History of medications/ dietary supplement**					
Hormones	2,374 (47.9%)	1,467 (49.7%)	816 (46.5%)	91 (36.0%)	**0.003**
Glucocorticosteroid	357 (6.1%)	171 (4.4%)	164 (8.6%)	22 (7.8%)	**<0.001**
Dietary supplement	2,426 (46.9%)	1,486 (47.5%)	824 (45.5%)	116 (49.0%)	0.480
**VD3 (nmol/L)**	65.71 (± 26.28)	63.59 (± 26.43)	69.63 (± 25.81)	62.65 (± 24.66)	**<0.001**
**ALP (IU/L)**	74.83 (± 24.44)	74.16 (± 24.01)	75.02 (± 24.02)	81.59 (± 30.84)	0.321
**Femoral Neck T-score**	-0.90 (± 0.93)	-0.28 (± 0.53)	-1.65 (± 0.42)	-2.91 (± 0.41)	**<0.001**

Abbreviations: PIR, poverty income ratio. BMI, body mass index. CKD, chronic kidney disease. VD3, 25-hydroxyvitamin D3. ALP, alkaline phosphatase. BMD, bone mineral density. NHANES, National Health and Nutrition Examination Survey.

Characterization statistics are presented as weighted mean (± weighted SD) or unweighted sample size (N) (weighted %). SD, standard deviation.

P-values for continuous variables were calculated using weighted analysis of variance (ANOVA), and P-values for categorical variables were calculated using weighted chi-square tests.

Table S3 Associations of age at menopause with Femoral Neck T-score.

Age at menopause, (year)	Model 1		Model 2		Model 3		Model 4		Model 5	
	β (95% CI)	P-value	β (95% CI)	P-value	β (95% CI)	P-value	β (95% CI)	P-value	β (95% CI)	P-value
Continuous variable	< 0.001 (-0.004, 0.004)	0.845	< 0.001 (-0.004, 0.004)	0.964	0.003 (-0.001, 0.008)	0.156	0.003 (-0.002, 0.008)	0.234	0.004 (-0.001, 0.007)	0.112
Grouped variable (Median [Q1, Q3])										
Q1 (35 [32, 39])	Reference		Reference		Reference		Reference		Reference	
Q2 (45 [44, 47])	-0.006 (-0.096, 0.083)	0.892	-0.001 (-0.092, 0.090)	0.985	0.026 (-0.069, 0.121)	0.596	0.031 (-0.062, 0.124)	0.512	0.068 (-0.025, 0.161)	0.154
Q3 (50 [50, 52])	-0.015 (-0.100, 0.070)	0.727	-0.018 (-0.108, 0.072)	0.695	0.041 (-0.060, 0.142)	0.428	0.040 (-0.062, 0.142)	0.444	0.079 (-0.020, 0.178)	0.122
Q4 (55 [54, 56])	-0.007 (-0.108, 0.093)	0.886	-0.001 (-0.104, 0.102)	0.983	0.064 (-0.043, 0.170)	0.243	0.056 (-0.051, 0.162)	0.308	0.082 (-0.022, 0.185)	0.124
P for trend	0.816		0.870		0.238		0.306		0.105	

Model 1: did not adjusted for relevant covariates. Model 2: adjusted for age, race, marital, education, PIR, smoking, drinking, physical activity. Model 3: further adjusted for age at menarche, pregnancy, delivery, hysterectomy, ovariectomy. Model 4: further adjusted for diabetes, osteoarthritis, rheumatoid arthritis, CKD, hormones, glucocorticoids, anti-osteoporosis medication, dietary supplements. Model 5: further adjusted for BMI, VD3, ALP.

P for trend were calculated by modeling the median values of each quantile.

Table S4 Associations of age at menopause with Femoral Neck T-score (unweighted).

Age at menopause, (year)	Model 1		Model 2		Model 3		Model 4		Model 5	
	β (95% CI)	P-value	β (95% CI)	P-value	β (95% CI)	P-value	β (95% CI)	P-value	β (95% CI)	P-value
Continuous variable	< 0.001 (-0.003, 0.003)	0.856	0.001 (-0.002, 0.004)	0.413	0.005 (0.001, 0.008)	**0.006**	0.004 (0.001, 0.008)	**0.023**	0.004 (0.001,0.007)	**0.019**
Grouped variable (Median [Q1, Q3])										
Q1 (35 [31, 39])	Reference		Reference		Reference		Reference		Reference	
Q2 (45 [44, 47])	-0.031 (-0.099, 0.037)	0.375	-0.015 (-0.081, 0.005)	0.649	0.015 (-0.053, 0.084)	0.662	0.011 (-0.056, 0.079)	0.743	0.034 (-0.031, 0.099)	0.301
Q3 (50 [50, 52])	-0.002 (-0.071, 0.068)	0.964	0.015 (-0.053, 0.082)	0.668	0.083 (0.007, 0.158)	**0.033**	0.074 (-0.001, 0.148)	0.053	0.092 (0.021, 0.164)	**0.011**
Q4 (55 [54, 56])	-0.014 (-0.089, 0.061)	0.720	0.017 (-0.055, 0.090)	0.644	0.091 (0.009, 0.172)	**0.030**	0.075 (-0.005, 0.155)	0.066	0.072 (-0.005, 0.148)	0.069
P for trend	0.822		0.566		**0.015**		**0.035**		**0.024**	

Model 1: did not adjusted for relevant covariates. Model 2: adjusted for age, race, marital, education, PIR, smoking, drinking, physical activity. Model 3: further adjusted for age at menarche, pregnancy, delivery, hysterectomy, ovariectomy. Model 4: further adjusted for diabetes, osteoarthritis, rheumatoid arthritis, CKD, hormones, glucocorticoids, anti-osteoporosis medication, dietary supplements. Model 5: further adjusted for BMI, VD3, ALP.

P for trend were calculated by modeling the median values of each quantile.

Table S5 Association between age at menopause and osteoporosis risk (unweighted).

Age at menopause, (years)	Model 1		Model 2		Model 3		Model 4		Model 5	
	OR (95% CI)	P-value	OR (95% CI)	P-value	OR (95% CI)	P-value	OR (95% CI)	P-value	OR (95% CI)	P-value
Continuous variable	0.994 (0.980, 1.008)	0.372	0.995 (0.980, 1.009)	0.468	0.991 (0.974, 1.008)	0.291	0.991 (0.974, 1.008)	0.314	0.992 (0.974, 1.011)	0.407
Grouped variable (Median [Q1, Q3])										
Q1 (35 [32, 39])	Reference		Reference		Reference		Reference		Reference	
Q2 (45 [44, 47])	0.821 (0.602, 1.120)	0.213	0.828 (0.602, 1.140)	0.247	0.801 (0.572, 1.120)	0.195	0.787 (0.561, 1.103)	0.165	0.757 (0.532, 1.079)	0.124
Q3 (50 [50, 52])	0.683 (0.489, 0.953)	**0.025**	0.706 (0.501, 0.995)	**0.047**	0.642 (0.438, 0.941)	**0.023**	0.622 (0.425, 1.912)	**0.015**	0.596 (0.398, 0.892)	**0.012**
Q4 (55 [54, 56])	0.937 (0.673, 1.306)	0.701	0.971 (0.688, 1.371)	0.867	0.867 (0.588, 1.278)	0.470	0.867 (0.588, 1.278)	0.472	0.882 (0.586, 1.329)	0.549
P for trend	0.238		0.367		0.198		0.179		0.217	

Model 1: did not adjusted for relevant covariates. Model 2: adjusted for age, race, marital, education, PIR, smoking, drinking, physical activity. Model 3: further adjusted for age at menarche, pregnancy, delivery, hysterectomy, ovariectomy. Model 4: further adjusted for diabetes, osteoarthritis, rheumatoid arthritis, CKD, hormones, glucocorticoids, anti-osteoporosis medication, dietary supplements. Model 5: further adjusted for BMI, VD3, ALP.

P for trend were calculated by modeling the median values of each quantile.

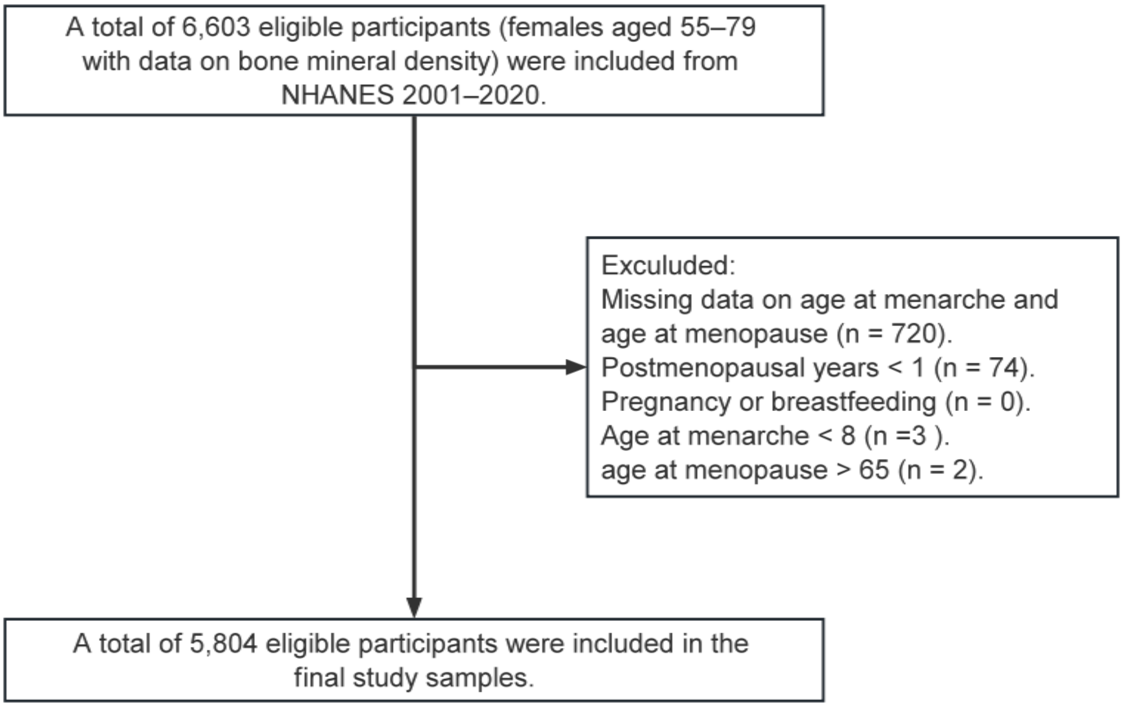

Fig. S1 Study population selection chart. Abbreviations: NHANES, National Health and Nutrition Examination Survey.

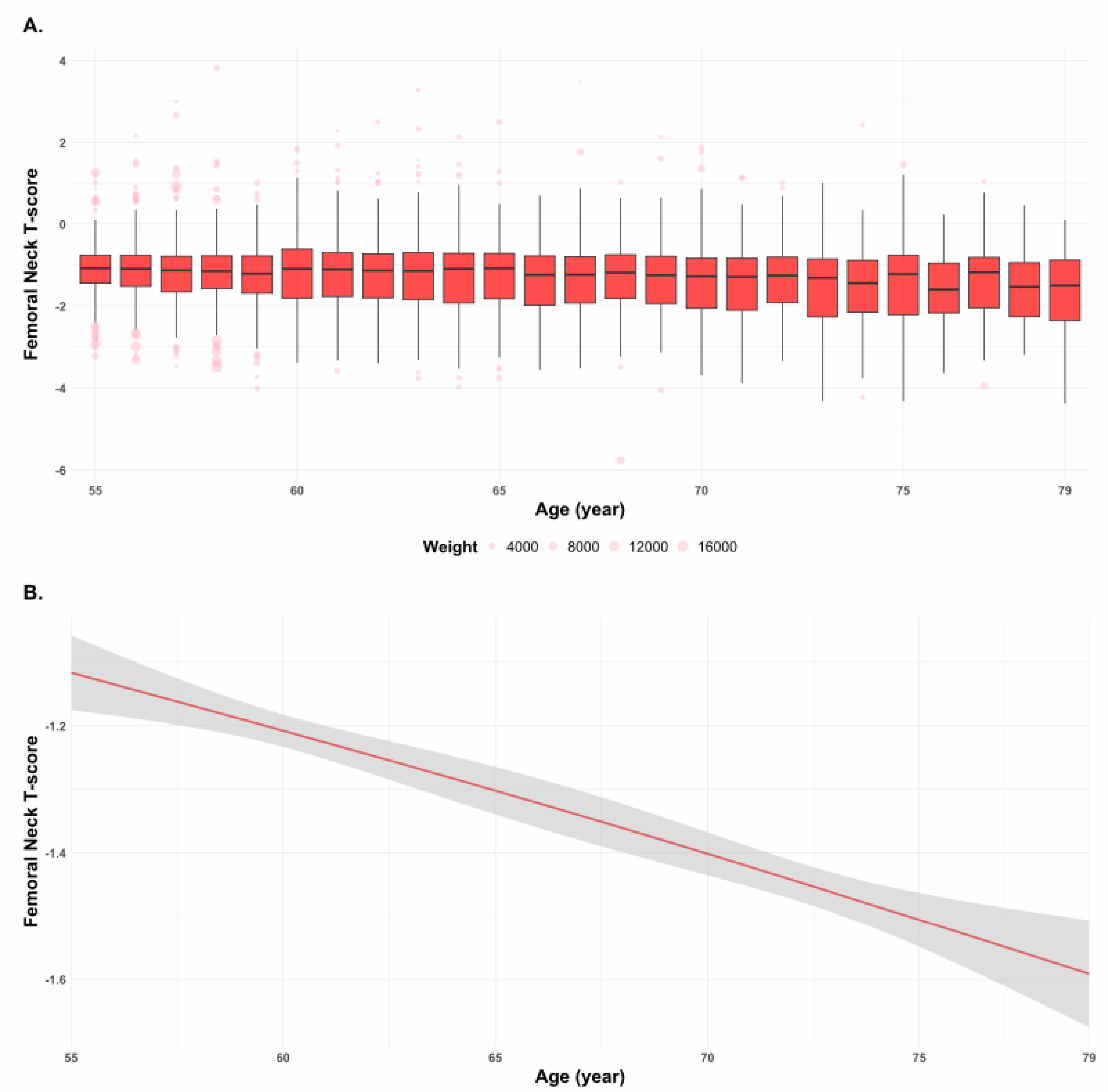

Fig. S2 Association between Age and Femoral Neck T-Score. (A) Box-and-whisker plots display the distribution of femoral neck T-scores across age groups, with scatter plot sizes scaled proportionally to the weighted sample size. (B) A weighted generalized additive model (weighted GAM) was used to fit the scatter plot of the overall population, revealing the trend of femoral neck T-scores with age. The shaded area represents the 95% CI.

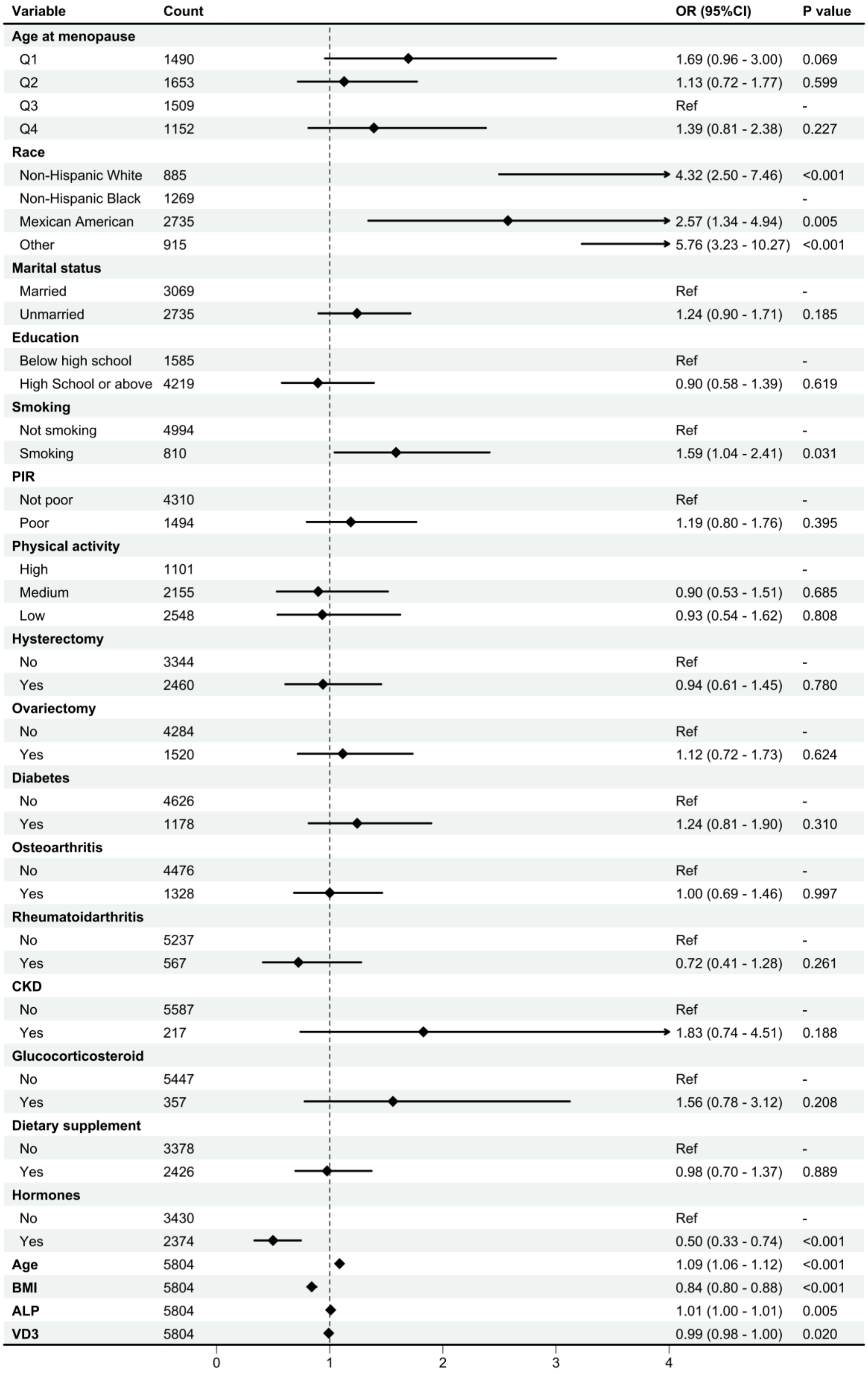

Variable	Count	OR (95%CI)	P value
**Age at menopause**			
Q1	1490	1.69 (0.96 - 3.00)	0.069
Q2	1653	1.13 (0.72 - 1.77)	0.599
Q3	1509	Ref	-
Q4	1152	1.39 (0.81 - 2.38)	0.227
**Race**			
Non-Hispanic White	885	4.32 (2.50 - 7.46)	<0.001
Non-Hispanic Black	1269		-
Mexican American	2735	2.57 (1.34 - 4.94)	0.005
Other	915	5.76 (3.23 - 10.27)	<0.001
**Marital status**			
Married	3069	Ref	-
Unmarried	2735	1.24 (0.90 - 1.71)	0.185
**Education**			
Below high school	1585	Ref	-
High School or above	4219	0.90 (0.58 - 1.39)	0.619
**Smoking**			
Not smoking	4994	Ref	-
Smoking	810	1.59 (1.04 - 2.41)	0.031
**PIR**			
Not poor	4310	Ref	-
Poor	1494	1.19 (0.80 - 1.76)	0.395
**Physical activity**			
High	1101		-
Medium	2155	0.90 (0.53 - 1.51)	0.685
Low	2548	0.93 (0.54 - 1.62)	0.808
**Hysterectomy**			
No	3344	Ref	-
Yes	2460	0.94 (0.61 - 1.45)	0.780
**Ovariectomy**			
No	4284	Ref	-
Yes	1520	1.12 (0.72 - 1.73)	0.624
**Diabetes**			
No	4626	Ref	-
Yes	1178	1.24 (0.81 - 1.90)	0.310
**Osteoarthritis**			
No	4476	Ref	-
Yes	1328	1.00 (0.69 - 1.46)	0.997
**Rheumatoidarthritis**			
No	5237	Ref	-
Yes	567	0.72 (0.41 - 1.28)	0.261
**CKD**			
No	5587	Ref	-
Yes	217	1.83 (0.74 - 4.51)	0.188
**Glucocorticosteroid**			
No	5447	Ref	-
Yes	357	1.56 (0.78 - 3.12)	0.208
**Dietary supplement**			
No	3378	Ref	-
Yes	2426	0.98 (0.70 - 1.37)	0.889
**Hormones**			
No	3430	Ref	-
Yes	2374	0.50 (0.33 - 0.74)	<0.001
**Age**	5804	1.09 (1.06 - 1.12)	<0.001
**BMI**	5804	0.84 (0.80 - 0.88)	<0.001
**ALP**	5804	1.01 (1.00 - 1.01)	0.005
**VD3**	5804	0.99 (0.98 - 1.00)	0.020

Fig. S3 Odds ratios (ORs) for osteoporosis risk factors. The estimated are based on a logistic regression model fully adjusted for all prespecified covariates. The centers of the diamonds in the figure correspond to the OR estimates for each subgroup. The horizontal lines extending from the diamond points indicate the 95% confidence intervals (CI); the vertical dashed lines mark the null line OR = 1. Arrows indicate confidence intervals extending beyond the coordinate axes.

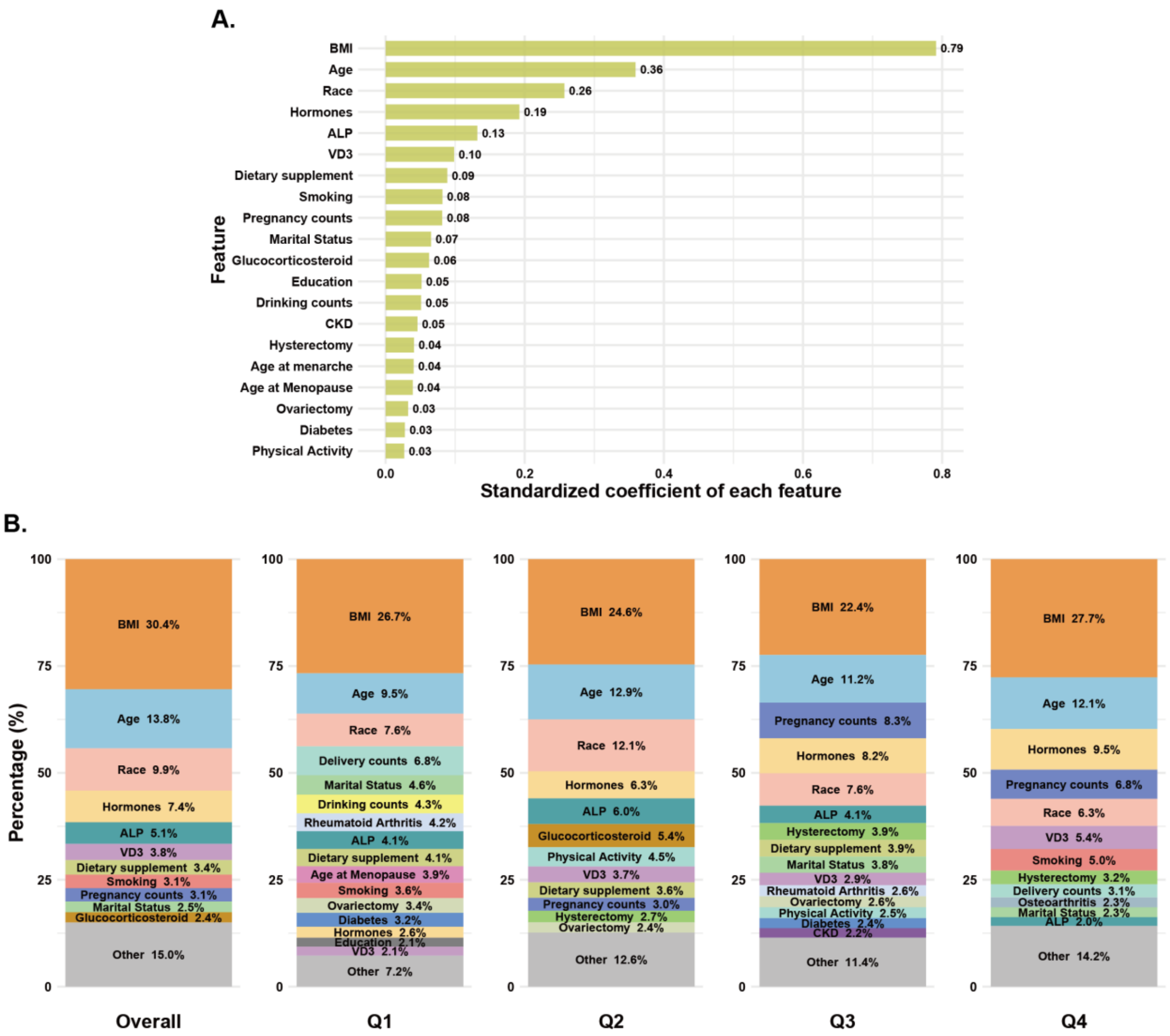

Fig. S4 Visualization of standardized coefficient calculation. (A) Top 20 variables ranked by and standardized coefficients. (B) Relative standardized coefficient importance of each variable. Based on the fully adjusted logistic regression model (Model 5).

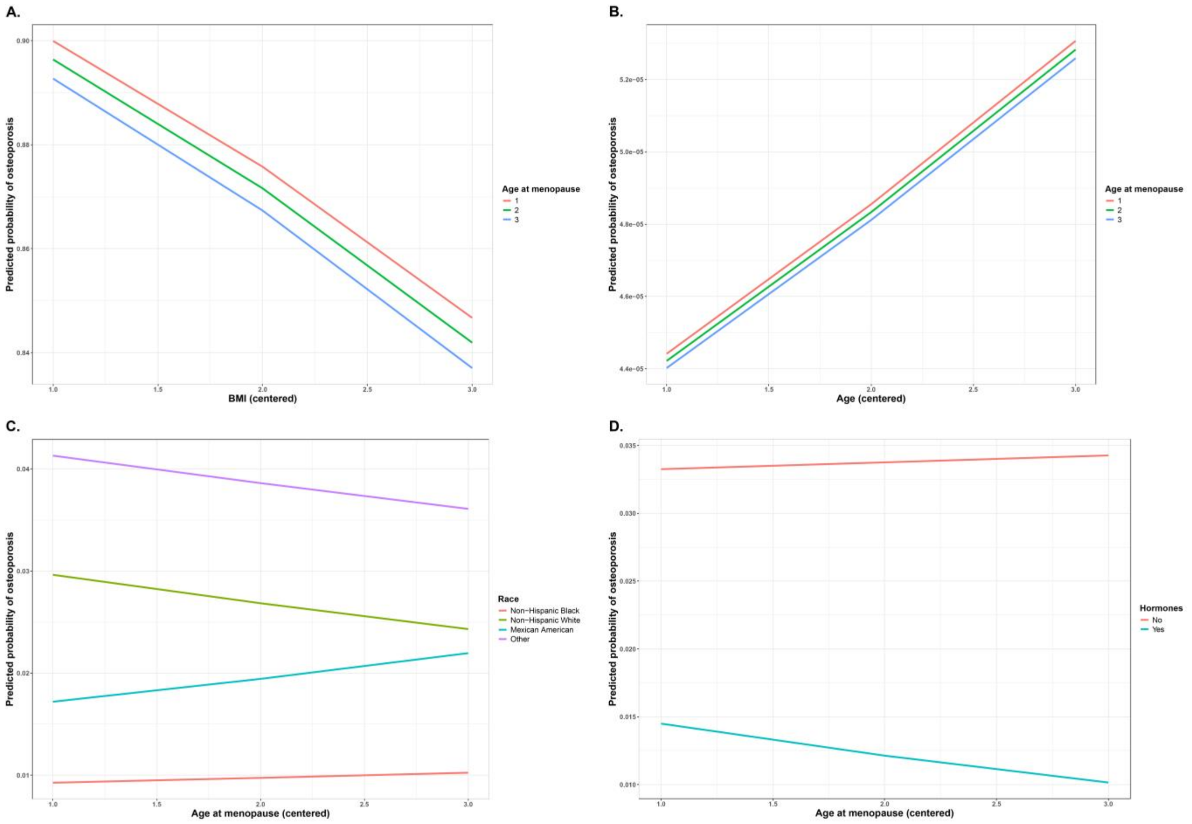

Fig. S5 Interaction effect plot. (A) Interaction effect between BMI and age at menopause. (B) Interaction effect between hormone use and age at menopause. (C) Interaction effect between BMI and hormone use.

Variable	Count	OR(95%CI)	P value	P for interaction
**Overall**	5804	0.54 (0.40 - 0.72)	<0.001	
**Age**				0.217
≤65	3273	0.37 (0.21 - 0.65)	0.001	
>65	2531	0.59 (0.41 - 0.86)	0.006	
**Age at menopause**				0.365
<40	1211	0.67 (0.34 - 1.33)	0.256	
40-50	2869	0.48 (0.30 - 0.76)	0.002	
>50	1724	0.43 (0.24 - 0.79)	0.007	
**Race**				0.477
Other	915	0.82 (0.42 - 1.63)	0.576	
Mexican American	885	0.37 (0.10 - 1.37)	0.138	
Non-Hispanic Black	1269	0.19 (0.03 - 1.36)	0.098	
Non-Hispanic White	2735	0.56 (0.39 - 0.82)	0.003	
**Marital status**				0.798
Married	3069	0.57 (0.36 - 0.90)	0.015	
Unmarried	2735	0.51 (0.34 - 0.78)	0.002	
**Education**				0.766
Below high school	1585	0.59 (0.32 - 1.09)	0.095	
High School or above	4219	0.51 (0.36 - 0.73)	<0.001	
**PIR**				0.361
Poor	1494	0.57 (0.31 - 1.04)	0.069	
Not poor	4310	0.53 (0.37 - 0.76)	<0.001	
**Smoking**				0.477
Smoking	810	0.56 (0.27 - 1.16)	0.121	
Not smoking	4994	0.52 (0.37 - 0.74)	<0.001	
**Physical activity**				0.073
High	1101	0.93 (0.42 - 2.05)	0.858	
Medium	2155	0.35 (0.21 - 0.59)	<0.001	
Low	2548	0.60 (0.38 - 0.94)	0.025	
**Hysterectomy**				0.004
Yes	2460	0.83 (0.53 - 1.29)	0.401	
No	3344	0.33 (0.20 - 0.53)	<0.001	
**Ovariectomy**				0.048
Yes	1520	0.85 (0.47 - 1.55)	0.592	
No	4284	0.42 (0.29 - 0.62)	<0.001	
**BMI**				0.074
<25	1383	0.46 (0.30 - 0.72)	0.001	
25-29.9	1855	0.69 (0.39 - 1.20)	0.189	
30-34.9	1345	0.34 (0.13 - 0.89)	0.029	
≥35	1221	0.53 (0.06 - 4.51)	0.563	
**Diabetes**				0.088
Yes	1178	0.68 (0.31 - 1.48)	0.33	
No	4626	0.49 (0.35 - 0.68)	<0.001	
**Osteoarthritis**				0.054
Yes	1328	0.33 (0.17 - 0.66)	0.002	
No	4476	0.66 (0.47 - 0.93)	0.018	
**Rheumatoidarthritis**				0.186
Yes	567	0.43 (0.11 - 1.67)	0.222	
No	5237	0.55 (0.41 - 0.76)	<0.001	
**CKD**				0.688
Yes	217	0.04 (0.00 - 0.64)	0.023	
No	5587	0.55 (0.41 - 0.76)	<0.001	
**Glucocorticosteroid**				0.653
Yes	357	0.08 (0.01 - 0.75)	0.027	
No	5447	0.54 (0.40 - 0.74)	<0.001	
**Anti-osteoporosis medication**				0.128
Yes	722	0.61 (0.35 - 1.07)	0.085	
No	5082	0.48 (0.33 - 0.70)	<0.001	
**Dietary supplement**				0.032
Yes	2426	0.43 (0.27 - 0.69)	<0.001	
No	3378	0.64 (0.43 - 0.96)	0.031	

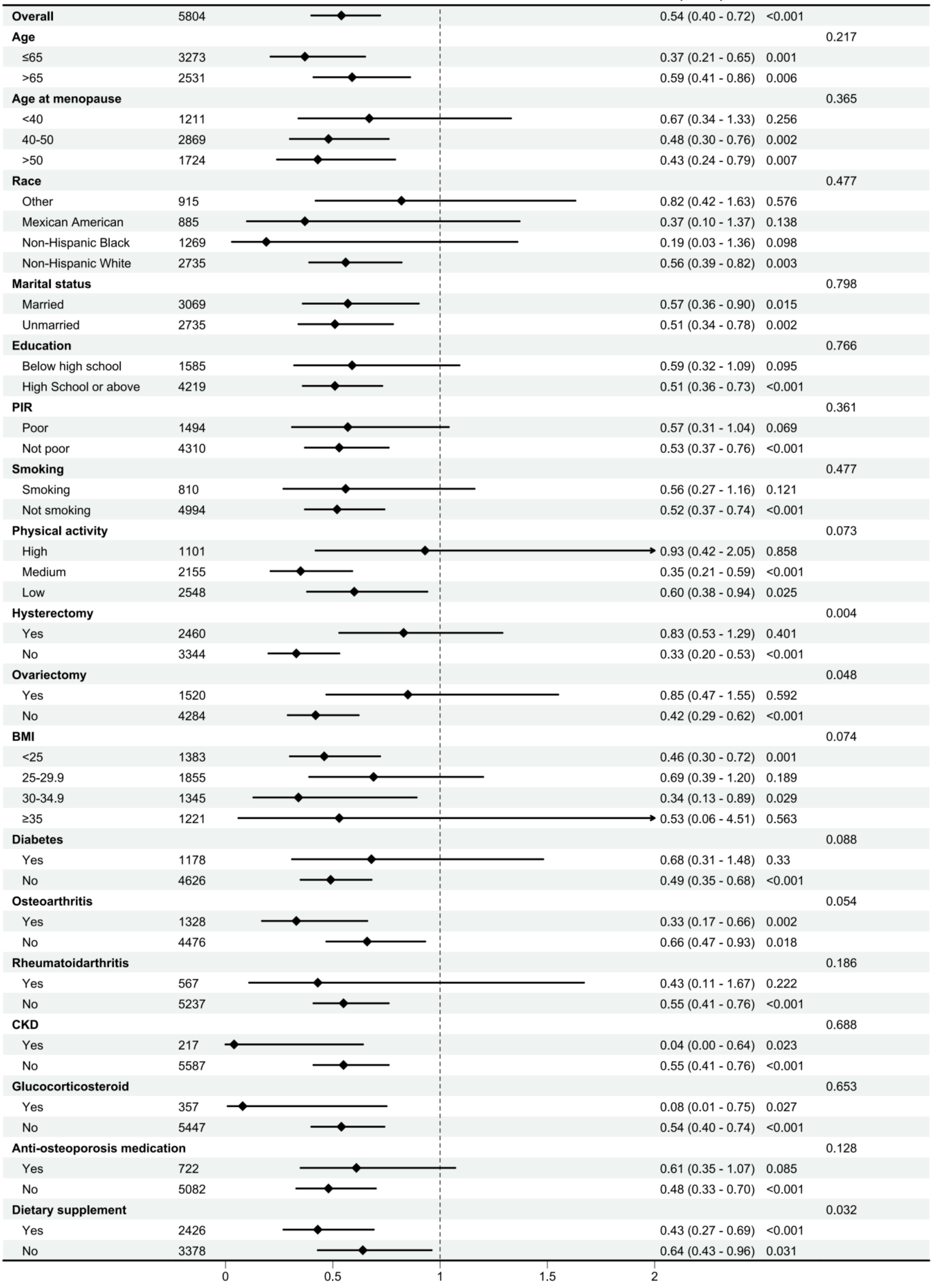

Fig. S6. Subgroup analyses of the association between hormone-use history and osteoporosis risk. The estimated are based on a logistic regression model fully adjusted for all prespecified covariates. The centers of the diamonds in the figure correspond to the OR estimates for each subgroup. The horizontal lines extending from the diamond points indicate the 95% confidence intervals (CI); the vertical dashed lines mark the null line OR = 1. Arrows indicate confidence intervals extending beyond the coordinate axes.

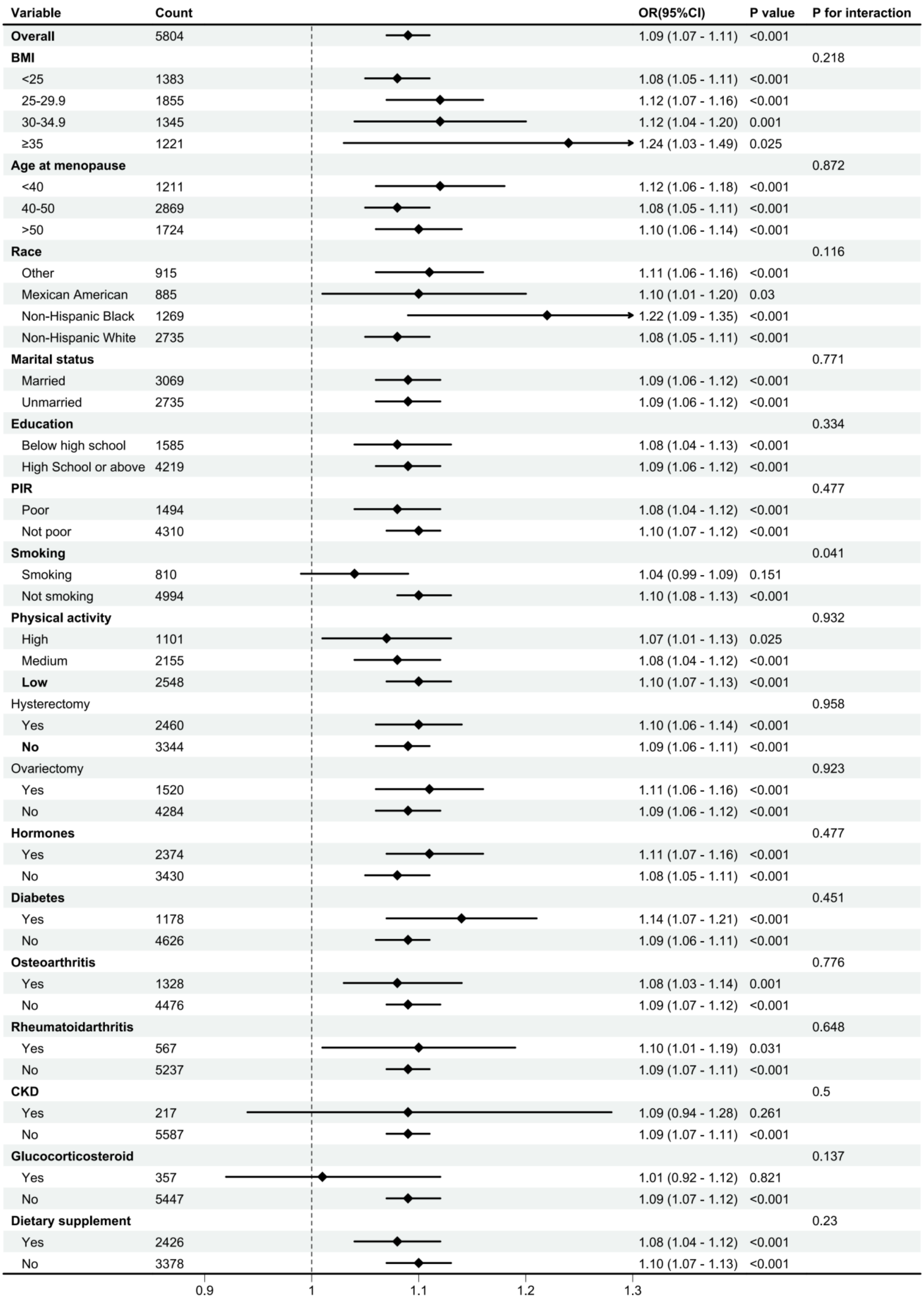

Fig. S7. Subgroup analyses of the association between age and osteoporosis risk. The estimated are based on a logistic regression model fully adjusted for all prespecified covariates. The centers of the diamonds in the figure correspond to the OR estimates for each subgroup. The horizontal lines extending from the diamond points indicate the 95% confidence intervals (CI); the vertical dashed lines mark the null line OR = 1. Arrows indicate confidence intervals extending beyond the coordinate axes.

Variable	Count	OR(95%CI)	P value	P for interaction
**Overall**	5804	0.99 (0.98 - 1.01)	0.537	
**Age**				0.96
≤65	3273	1.00 (0.97 - 1.03)	0.981	
>65	2531	0.99 (0.97 - 1.02)	0.59	
**BMI**				0.676
<25	1383	0.99 (0.96 - 1.01)	0.276	
25-29.9	1855	0.98 (0.95 - 1.02)	0.276	
30-34.9	1345	1.02 (0.96 - 1.08)	0.556	
≥35	1221	1.04 (0.94 - 1.15)	0.446	
**Race**				0.755
Other	915	0.98 (0.94 - 1.03)	0.428	
Mexican American	885	1.06 (0.98 - 1.14)	0.149	
Non-Hispanic Black	1269	0.95 (0.88 - 1.04)	0.257	
Non-Hispanic White	2735	0.99 (0.97 - 1.02)	0.607	
**Marital status**				0.49
Married	3069	1.00 (0.97 - 1.03)	0.884	
Unmarried	2735	0.99 (0.96 - 1.01)	0.276	
**Education**				0.872
Below high school	1585	0.97 (0.94 - 1.01)	0.135	
High School or above	4219	1.00 (0.98 - 1.03)	0.859	
**PIR**				0.814
Poor	1494	1.00 (0.97 - 1.03)	0.957	
Not poor	4310	0.99 (0.97 - 1.01)	0.464	
**Smoking**				0.501
Smoking	810	0.97 (0.93 - 1.02)	0.274	
Not smoking	4994	1.00 (0.98 - 1.02)	0.952	
**Physical activity**				0.597
High	1101	1.02 (0.97 - 1.07)	0.44	
Medium	2155	1.00 (0.97 - 1.03)	0.823	
Low	2548	0.99 (0.96 - 1.01)	0.334	
**Hysterectomy**				0.684
Yes	2460	0.99 (0.97 - 1.02)	0.691	
No	3344	1.00 (0.97 - 1.03)	0.938	
**Ovariectomy**				0.903
Yes	1520	1.00 (0.97 - 1.04)	0.883	
No	4284	0.99 (0.97 - 1.02)	0.53	
**Hormones**				0.145
Yes	2374	0.99 (0.96 - 1.02)	0.709	
No	3430	1.00 (0.97 - 1.02)	0.723	
**Diabetes**				0.881
Yes	1178	0.99 (0.95 - 1.04)	0.79	
No	4626	0.99 (0.97 - 1.01)	0.461	
**Osteoarthritis**				0.866
Yes	1328	0.99 (0.95 - 1.04)	0.704	
No	4476	0.99 (0.97 - 1.01)	0.554	
**Rheumatoidarthritis**				0.222
Yes	567	1.03 (0.96 - 1.11)	0.418	
No	5237	0.99 (0.97 - 1.01)	0.339	
**CKD**				0.835
Yes	217	0.94 (0.85 - 1.04)	0.236	
No	5587	1.00 (0.98 - 1.01)	0.617	
**Glucocorticosteroid**				0.865
Yes	357	0.99 (0.90 - 1.09)	0.829	
No	5447	0.99 (0.97 - 1.01)	0.421	
**Dietary supplement**				0.305
Yes	2426	1.00 (0.97 - 1.04)	0.749	
No	3378	0.99 (0.97 - 1.01)	0.37	

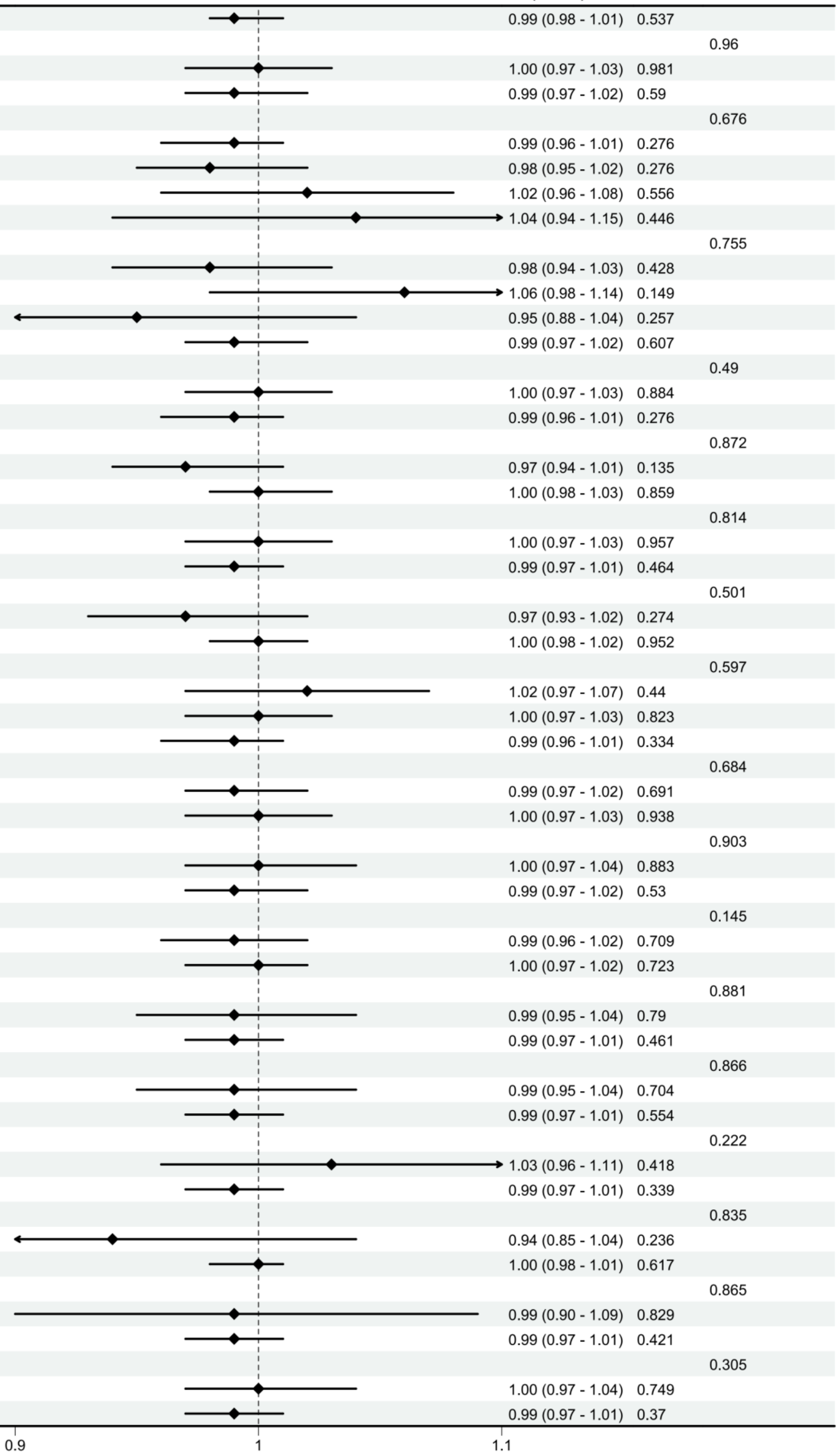

Fig. S8. Subgroup analyses of the association between age at menopause and osteoporosis risk. The estimated are based on a logistic regression model fully adjusted for all prespecified covariates. The centers of the diamonds in the figure correspond to the OR estimates for each subgroup. The horizontal lines extending from the diamond points indicate the 95% confidence intervals (CI); the vertical dashed lines mark the null line OR = 1. Arrows indicate confidence intervals extending beyond the coordinate axes.

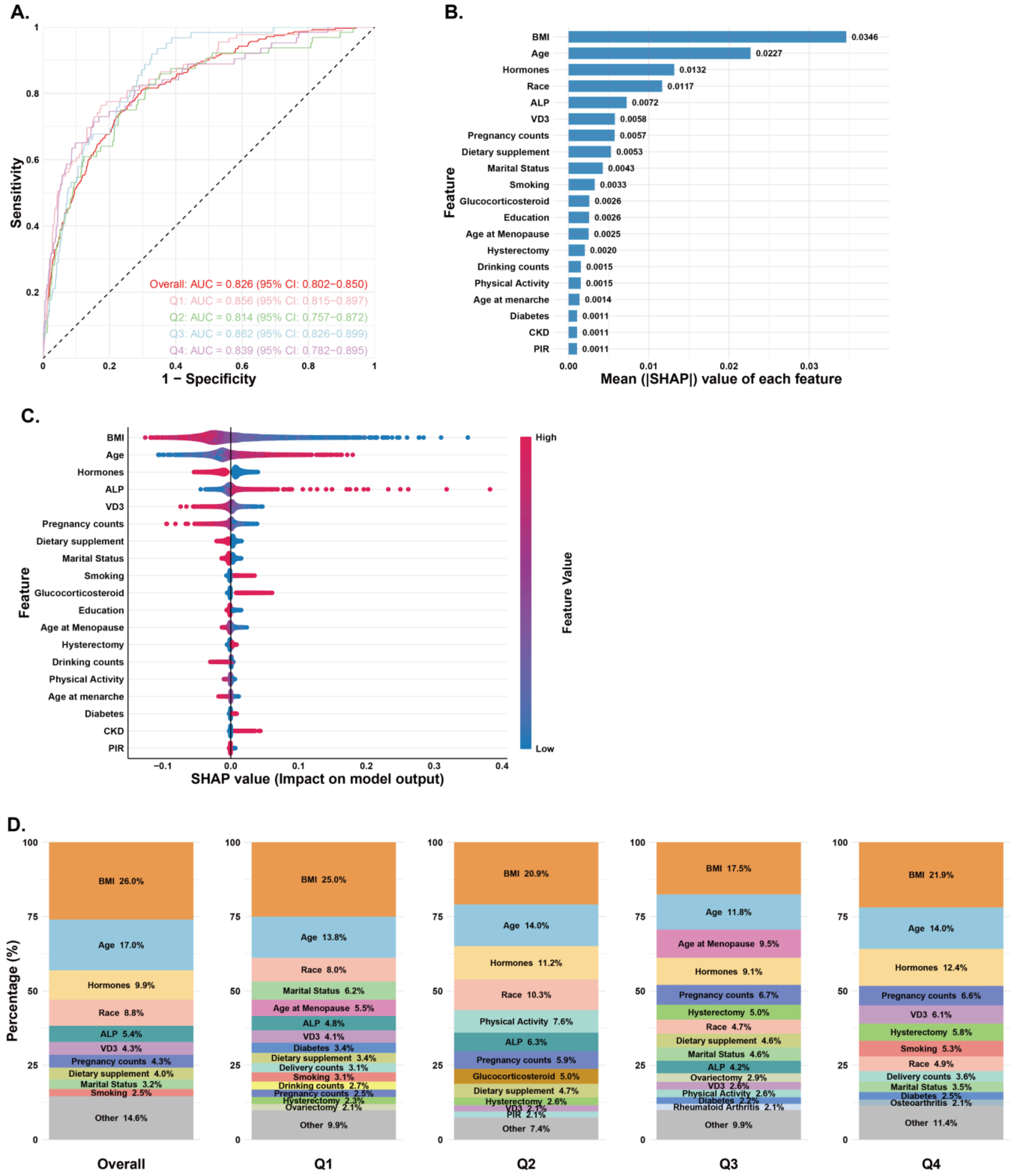

Fig. S9 Sensitivity analyses (excluding participants with extreme age at menopause). (A) Receiver Operating Characteristic (ROC) curves for the model across all groups. (B) Top 20 variables ranked by SHAP values. (C) SHAP summary plot. (D) Relative SHAP importance of each variable. Analysis based on the fully adjusted logistic regression model (Model 5).

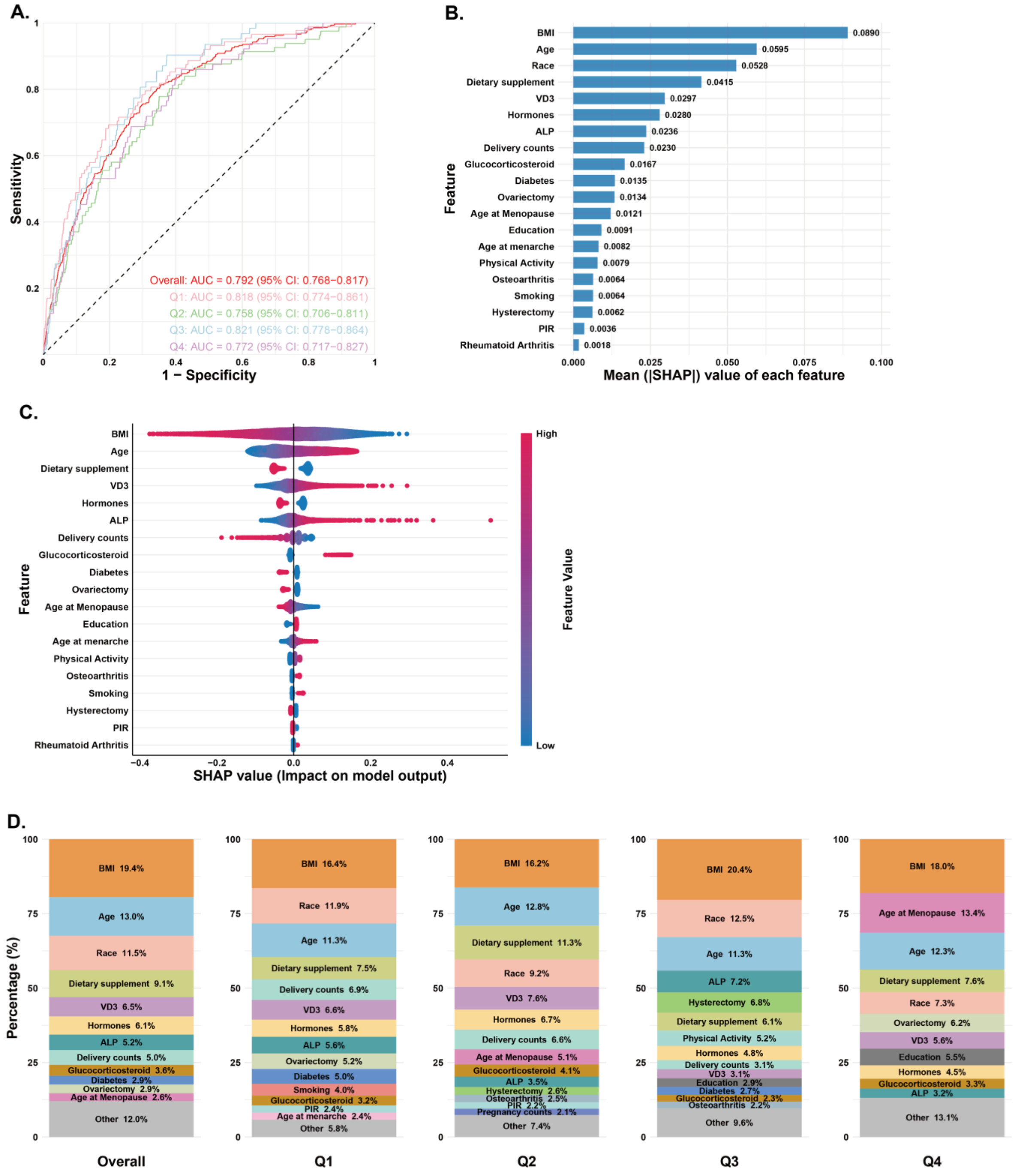

Fig. S10 Sensitivity analyses (outcome was restricted to osteopenia). (A) Receiver Operating Characteristic (ROC) curves for the model across all groups. (B) Top 20 variables ranked by SHAP values. (C) SHAP summary plot. (D) Relative SHAP importance of each variable. Analysis based on the fully adjusted logistic regression model (Model 5).
